# A Pharmacological Overview of Alpinumisoflavone, a Natural Prenylated Isoflavonoid

**DOI:** 10.3389/fphar.2019.00952

**Published:** 2019-09-10

**Authors:** Sylvin Benjamin Ateba, Marie Alfrede Mvondo, Sefirin Djiogue, Stéphane Zingué, Liselotte Krenn, Dieudonné Njamen

**Affiliations:** ^1^Laboratory of Animal Physiology, Department of Animal Biology and Physiology, Faculty of Science, University of Yaoundé I, Yaoundé, Cameroon; ^2^Research Unit of Animal Physiology and Phytopharmacology, Department of Animal Biology, Faculty of Science, University of Dschang, Dschang, Cameroon; ^3^Department of Life and Earth Sciences, Higher Teachers’ Training College, University of Maroua, Maroua, Cameroon; ^4^Department of Pharmacognosy, University of Vienna, Vienna, Austria

**Keywords:** alpinumisoflavone, therapeutic potential, natural product, prenylated isoflavonoid, structure–activity relationship

## Abstract

Over the last decade, several studies demonstrated that prenylation of flavonoids enhances various biological activities as compared to the respective nonprenylated compounds. In line with this, the natural prenylated isoflavonoid alpinumisoflavone (AIF) has been explored for a number of biological and pharmacological effects (therapeutic potential). In this review, we summarize the current information on health-promoting properties of AIF. Reported data evidenced that AIF has a multitherapeutic potential with antiosteoporotic, antioxidant and anti-inflammatory, antimicrobial, anticancer, estrogenic and antiestrogenic, antidiabetic, and neuroprotective properties. However, research on these aspects of AIF is not sufficient and needs to be reevaluated using more appropriate methods and methodology. Further series of studies are needed to confirm these pharmacological effects, and this review should lay the basis for the design of respective investigations. Overall, despite the drawbacks of studies recorded, AIF exhibits a potential as drug candidate.

## Introduction

In the drug discovery process, plants still remain an invaluable source of drugs and drug leads. They possess enormous structural and chemical diversity that is not matched by any synthetic libraries of small molecules (Shen, 2015). As pharmacological activities of chemicals are generally structure dependent, the structural and chemical diversity is obviously an advantage. Over the last decade, the interest in (iso)flavonoids strongly increased. Especially the prenylated forms moved into the focus because of their versatile and promising pharmacological properties and health benefits on multitarget tissues (Kumar and Pandey, 2013; Chen et al., 2014). Prenylated isoflavonoids have increased lipophilicity as compared to nonprenylated forms, leading to high affinity with cell membranes and enhanced biological activities or significant pharmacological effects (Chen et al., 2014; Sherif et al., 2015). These compounds offer a multitude of biological activities, which justify major and much deeper pharmacological investigation (Botta et al., 2009). Accordingly, there is a recent in-depth investigation of prenylated flavonoids as promising anticancer, anti-inflammatory, antioxidant, and neuroprotective nutraceuticals (Yang et al., 2015; Venturelli et al., 2016), with the prenyl substituent playing a key role in the molecular activity. Prenylated flavonoids are found predominantly in the Leguminosae family, although the phenyl-propanoid pathway—necessary for their production—is ubiquitously present in plants including nonleguminous families (Reynaud et al., 2005; Lapčík, 2007).

**Figure 1:**
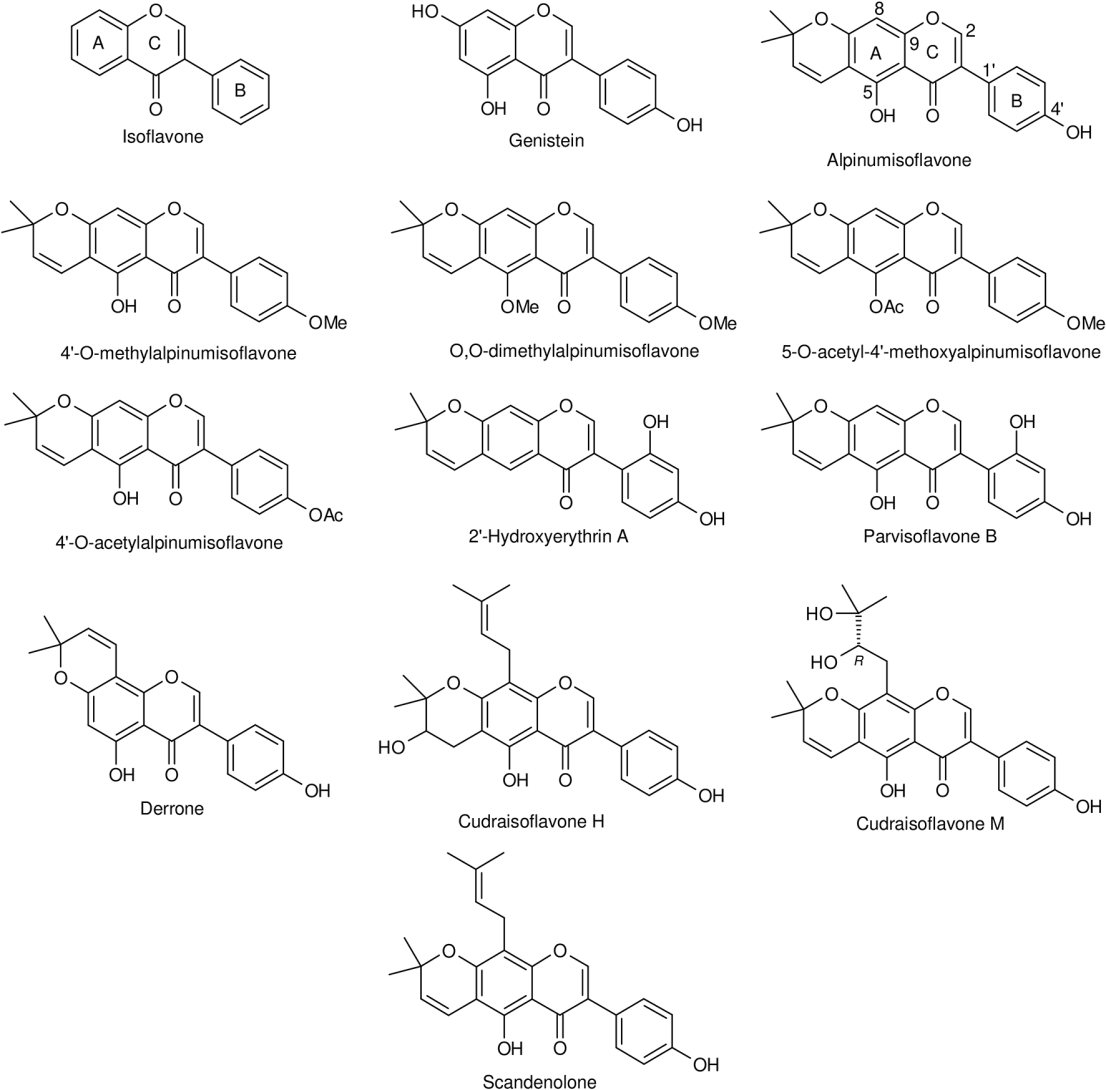
Chemical structures of isoflavone, genistein, alpinumisoflavone, and some of its derivatives.

Alpinumisoflavone (AIF) or [5-hydroxy-7-(p-hydroxyphenyl)-2,2-dimethyl-2H-6H-benzo-[1,2-b:5,4-b]dipyran-6-one] is a dimethylpyrano derivative prenylated at ring A of genistein (**Figure 1**). It is a major constituent of *Derris eriocarpa* F.C. How, commonly referred as “Tugancao” in “Zhuang” and “Dai” ethnomedicine in Guangxi and Yunnan Province of China (Guangxi Institute of Chinese Medicine, 1986). A high content of AIF was reported in fully mature fruits (mandarin melon berry) of *Cudrania tricuspidata* Bur. ex Lavallee (syn. *Maclura tricuspidata* Carrière) (Shin et al., 2015), a crop cultivated in East Asia (Xiong et al., 1993; Shi, 2010), Europe and America (Markovski, 2016) for its fruits and timber, and with an immense medicinal and economic value (Xin et al., 2017). Isolated for the first time by Jackson et al. (1971), AIF was identified in many medicinal plants widely used over the world (**Table 1**). Although data depicted in this table are not exhaustive, the global trend is in accordance with Botta et al. (2005) who reported that prenylated flavonoids occur mostly in Leguminosae and Moraceae, with few detected in other families. Over the last two decades, the body of literature of AIF and its pharmacological potential is steadily growing. This review summarizes and gives a critical look on the current knowledge of the biological activities, therapeutic potential, and mechanism of action of AIF.

**Table 1:** Sources and biological and/or pharmacological activities of alpinumisoflavone.

Family	Plant species	Plant parts	Origin/city (country)	Biological and/or pharmacological activity	References
**Leguminosae**	*Crotalaria bracteata*	Roots and stems	Roi-Et (Thailand)	Cytotoxicity against MCF-7 and NCI-H187 cell lines (inactive)	Sudanich et al., 2017
	*Erythrina caffra* Thunb.	Stem bark	KwaZulu-Natal (South Africa)	Anti-bacterial activity against *Staphylococcus aureus*, *Bacillus subtilis*, *Klebsiella pneumonia* and *Escherichia coli*	Chukwujekwu et al., 2011
	*Erythrina indica*	Stem bark	Ibadan (Nigeria)	*C*ytotoxicity against KB cells	Nkengfack et al., 2001
	*Erythrina lysistemon*	Stem bark	– (Zimbabwe)	Estrogen-like effects in a menopause model of ovariectomized Wistar rats	Mvondo et al., 2011; Mvondo et al., 2012; Mvondo et al., 2015
	*Erythrina mildbraedii*	Root bark	Buea (Cameroon)	Inhibition of protein tyrosine phosphatase-1B (PTP1B)	Na et al., 2006
	*Erythrina orientalis*	Stem bark	Kunir Kidul (Indonesia)	Cytotoxicity against murine leukemia P-388 cells Radical scavenging activity using DPPH (2,2-diphenyl-1-picrylhydrazyl)	Tjahjandarie and Tanjung, 2015a
	*Erythrina ovalifolia* Roxb.	Stem bark	Kunir Kidul (Indonesia)	Antiplasmodial activity against *Plasmodium falciparum*	Tjahjandarie and Tanjung, 2015b
	*Erythrina poeppigiana*	Stem bark	Sancta Cruz (Bolivia)	Estrogen-like effect in U2OS human osteosarcoma cells through ERs-dependent reporter gene activity	Djiogue et al., 2009; Djiogue et al., 2010
	*Erythrina senegalensis* DC	Stem bark	Foumban (Cameroon)	Inhibition of the HIV-1 Protease	Lee et al., 2009a
				Phospholipase Cγ1inhibitory activity (inactive) Inhibition of acyl CoA:diacylglycerol acyltransferase	Oh et al., 2005 Oh et al., 2009
	
	*Erythrina stricta* Roxb.	Stem bark	Nagaland (India)	Antimicrobial and radical scavenging (DPPH) activities	Akter et al., 2016
	*Erythrina suberosa* Roxb.	Stem bark	Jammu (India)	Cytotoxicity against human myeloid leukemia cell lines HL-60 and K-562 and T lymphoblastic cell line MOLT-4 Apoptotic potential in HL-60 cells	Kumar et al., 2013
	*Erythrina variegate* L.	Stem bark	Dhaka (Bangladesh)	Radical scavenging (DPPH) activity	Rahman et al., 2010
**Leguminosae**	*Derris eriocarpa*	–	–	Inhibition of osteoclast differentiation in vitro and antiosteoporotic effect in ovariectomized mice	Cong et al., 2017
		–	–	Suppression of tumor growth and metastasis of clear-cell renal cell carcinoma	Wang et al., 2017a
	*Laburnum alpinum* J. Presl.	Twigs	Salford (England)	–	Jackson et al., 1971
	*Lonchocarpus glabrescens*	–	Punchana, (Peru)	Inhibition of the hypoxia-inducible factor-1 (HIF-1) activation in human breast tumor T47D cells	Liu et al., 2009
	*Milletia pachycarpa*	Stem and leaves	–	No estrogenic activity on the β-galactosidase activity in a yeast two-hybrid assay	Okamoto et al., 2006
	*Millettia taiwaniana*	Twigs and leaves	– (Singapore)	Inhibition of the Epstein–Barr virus activation with no cytotoxicity against Raji cells	Ito et al., 2000
	*Millettia thonningii* (Schum. et Thonn.) Bak.	Seeds	Legon-Accra (Ghana)	Antifungal activity against wild-type *Candida albicans* and the reference strain ATCC18804C	Ayine-Tora et al., 2016
		Seeds	Accra (Ghana)	Antischistosomal activity against *Schistosoma mansoni*	Lyddiard et al., 2002
		Seeds	– (Ghana)	Antiplasmodial activity against *Plasmodium falciparum*	Khalid et al., 1986
	*Sophora moorcroftiana* (Wall.)	Aerial parts	Tibet (China)	Antibacterial effects on Meticillin-resistant *Staphylococcus aureus*	Wang et al., 2014
	*Tipuana tipu* (Benth.) Lillo	Leaves	Mansoura (Egypt)	Antiproliferative activity against leukemia [CCRF-CEM, MOLT-4, and HL-60(TB)], renal SN12C, and breast MCF-7 cancer cells Anti-inflammatory activity in carrageenan-induced rat paw edema model	Amen et al., 2013
**Moraceae**	*Chlorophora tinctoria* (L.) Gaud.	Leaves and twigs	Maynas (Peru)	Fatty acid synthase inhibitory and antifungal activities (inactive)	Li et al., 2002
	*Cudrania tricuspidata* (Carr.)	Fruits	Cheongju, (Korea)	Inhibition of the mouse brain monoamine oxidase	Han et al., 2005
		Twigs	Anhui (China)	Tyrosinase inhibition	Zheng et al., 2013
	*Ficus bengalensis*	Aerial roots	Sahiwal (Punjab, India)	–	Riaz et al., 2012
	*Ficus benjamina* var. nuda (Miq.) Barrett	Fruits	Honolulu (Hawaii)	–	Dai et al., 2012
	*Ficus chlamydocarpa* Mildbraed and Burret	Root bark	Bahouan (Cameroon)	Antimycobacterial, antibacterial and antifungal activities	Kuete et al., 2008
	*Ficus glumosa*	Stem bark	Makenene (Cameroon)	Cytotoxicity against prostate cancer PC-3 cell line	Nana et al., 2012
	*Ficus nervosa* Heyne ex Roth.		Pingtung (Taiwan)	–	Chen et al., 2010
	*Ficus racemosa*	Fruits	– (Vietnam)	Inhibition of protein tyrosine phosphatase-1B (PTP1B)	Trinh et al., 2017
	*Ficus tikoua* Bur	*Rhizomes*		Radical scavenging (DPPH) and α-glucosidase inhibitory activities	Fu et al., 2018
	*Maclura tricuspidata* Carrière (syn.*Cudrania tricuspidata*)	Fruits	Jinju (South Korea)	Cytotoxicity against human neuroblastoma SH-SY5Y cell line	Hong et al., 2018
**Dilleniaceae**	*Tetracera scandens*	Branch	– (Vietnam)	Glucose-uptake induced activity in basal and insulin-stimulated L6 myotubes	Lee et al., 2009b
**Apiaceae**	*Azorella madreporica*	Whole plant	Valle Nevado (Chile)	Antimycobacterial and antibacterial activities (inactive)	San-Martín et al., 2015

–‚ not indicated.

## Pharmacological Activities

Over the last decade research indicates that prenylation usually renders (iso)flavonoids with improved bioactivities (Yang et al., 2015; Mukai, 2018), suggesting that prenylated compounds have a higher potential to be developed and utilized (Chen et al., 2014). Focusing on AIF, the following activities have been demonstrated and claimed to be promising by the authors (**Table 2**).

**Table 2:** Pharmacological activities of alpinumisoflavone and underlying mechanisms.

Pharmacological activities	Experimental model	Dose/concentration	Mechanism of action	References
Estrogenic activity	ER competitor binding assay		Weak ERα and ERβ binder; higher selectivity for ERα	Mvondo et al., 2012; Magne Nde et al., 2012
	ER competitor binding assay		Weak ERα and ERβ binder; higher selectivity for ERβ	Djiogue et al., 2009
	U2OS-ERα, U2OS-ERβ human osteosarcoma cells	10^−9^–10^−6^ M	Induction of luciferase reporter gene activity	Djiogue et al., 2010; Magne Nde et al., 2012
	MCF-7 breast cancer cells	10^−9^–10^−6^ M	Up-regulation of the expression of estrogen α receptor target genes PCNA, cyclin D1, cyclinE1, cMyc, and LRH-1; downregulation of GREB1 at 10^−9^ M	Magne Nde et al., 2012
	Ovariectomized Wistar rats	0.01, 0.1, and 1 mg/kg daily for 3 days i.p.	Increase in uterine wet weight, and uterine and vaginal epithelial height	Mvondo et al., 2012
	Ovariectomized Wistar rats	1, 10 mg/kg daily for 28 days i.p.	Increase in uterine and vaginal epithelial height; increase in FSH/LH ratio; reduction in atherogenic risks	Mvondo et al., 2011
	Ovariectomized Wistar rats	0.1, 1, and 10 mg/kg daily for 3 days i.p.	Down-regulation of Esr1 mRNA expression; upregulation of Cyp7a1 mRNA expression.	Mvondo et al., 2015
Antiosteoporotic activity	RAW264.7 osteoclast precursor	2.5 and 5 µM	Suppression of osteoclast differentiation and proliferation by inhibiting RANKL-induced p38, ERK and JNK activation	Cong et al., 2017
	Ovariectomy-induced osteoporosis	10, 25 mg/kg daily for 6 weeks p.o.	Prevention of OVX-induced bone loss by increasing BV/TV ratio, Tb.Th and Tb.N while decreasing Tb.Sp in OVX mice	Cong et al., 2017
	Dexamethasone-induced osteoporosis	20, 40 mg/kg daily for 8 weeks p.o.	Increase in bone mineral density and mineral content of the proximal femur bone in rats; increase in BV/TV ratio, Tb.Th and Tb.N; decrease in Tb.Sp	Wang et al., 2017b
	MC3T3-E1 and MLO-Y4 osteoblasts and osteocytes	5–20 µM	Reverse of proapoptotic and antiproliferative effects of dexamethasone *via* suppressing Nox2-dependent ROS generation	Wang et al., 2017b; Yin et al., 2018
Antioxidant activity	DPPH assay	IC_50_: 8.30 µg/ml IC_50_: 708.5 µM IC_50_: 54.80 µg/ml IC_50_: 54.02 µg/ml	DPPH scavenging activity of differing degree	Rahman et al., 2010; Tjahjandarie and Tanjung, 2015a; Fu et al., 2018 Bórquez et al., 2013;
	Ferric Reducing Antioxidant Power (FRAP) assay	35.55 µM trolox equivalents/1.5 mM	Free radical-scavenging activity Increase in the FRAP reducing power	Bórquez et al., 2013;
	LPS-stimulated RAW264.7 cells	5, 10 µg/ml	Increase in catalase, HO-1, glutathione peroxidase, and superoxide dismutase production	Li et al., 2018
Anti-inflammatory activity	LPS-stimulated acute lung injury in mice	1, 5, 10 mg/kg i.p. 1 h before LPS challenge	Alleviated lung lesions, pulmonary edema, and hemorrhages: inhibition of myeloperoxidase activity	Li et al., 2018
	LPS-stimulated RAW264.7 cells	5, 10 µg/ml	Decreased production of TNF-α, IL-6, IL-1b, ICAM-1, and NO; suppression of NF-κB, MAPKs, and NLRP3 pathways	Li et al., 2018
	Carrageenan-induced rat paw edema	25 mg/kg i.p. 30 min before λ-carrageenan (unique dose)	Inhibition of edema formation	Amen et al., 2013
Antimicrobial activity	*Mycobacterium smegmatis* MC2 155	MIC = 19.53 µg/ml MBC = 39.06 µg/ml	Growth inhibition	Kuete et al., 2008
	*Enterobacter cloacae* LMP1104G *Escherichia coli* LMP0101U *Morganella morganii* LMP0904G *Proteus mirabilis* LMP0504G *Staphylococcus aureus* LMP0206U *Bacillus stearothermophilus* LMP0104G	IZ = 15.5–18.7 mm except for *E. coli* (7 mm) MIC = 39.06 µg/ml except for *P. mirabilis* (78.12 µg/ml)	Growth inhibition	Kuete et al., 2008
	*Candida albicans*	IZ = 14.5 mm MIC = 78.12 µg/ml	Growth inhibition	Kuete et al., 2008
	*Candida albicans* wild type *Candida albicans* ATCC18804	MIC = 0.25 µg/ml MIC = 0.50 µg/ml	Growth inhibition	Ayine-Tora et al., 2016
	3D structure of CdsD protein of Chlamydial T3SS		Interaction with the active site residue GLU-626(O-H) of contact-dependent secretion D (CdsD) protein	Sathishkumar and Tharani, 2017
	*Bacillus subtilis* ATCC6051 *Staphylococcus aureus* ATCC12600 *Klebsiella pneumoniae* ATCC 13883 *Escherichia coli* ATCC11775	MIC of 3.9 µg/ml except for *B. subtilis* (7.8 µg/ml)	Growth inhibition	Chukwujekwu et al., 2011
	*Staphylococcus aureus* SA1199B *Staphylococcus aureus* RN4220 *Staphylococcus aureus* EMRSA-15 *Staphylococcus aureus* XU212 *Staphylococcus aureus* EMRSA-16 *Staphylococcus aureus* ATCC25923	MIC = 64 µg/ml MIC = 128 µg/ml MIC = 128 µg/ml MIC > 128 µg/ml MIC > 128 µg/ml MIC > 128 µg/ml	Growth inhibition	Wang et al., 2014
Antimicrobial activity	*Staphylococcus aureus* MSSA *Staphylococcus aureus* MRSA *Staphylococcus aureus* MDRSA	MIC = 15 µg/ml MIC = 30 µg/ml MIC = 30 µg/ml	Growth inhibition	Akter et al., 2016
Anticancer activity	KB oral epidermoid carcinoma cells	ED_50_ = 4.13 µg/ml	Inhibition of cell proliferation	Nkengfack et al., 2001
	P-388 leukemia cells	IC_50_ = 4.31 µg/ml	Inhibition of cell proliferation	Tjahjandarie and Tanjung, 2015a
	HL-60, MOLT-4, K-562 leukemia cells	50 µM	Inhibition of cell proliferation; induction of apoptosis *via* both intrinsic and extrinsic pathways (activation of caspase-3, -8, -9; PARP cleavage; release of cytochrome c, Bax; downregulation of Bcl-2 expression) and inhibition of NF-kB (p65)/Stat3 tango in HL-60 cells	Kumar et al., 2013
	Full NCI 60 cell panel	10^−5^ M	Inhibition of proliferation of CCRF-CEM, MOLT-4, and HL-60(TB) leukemia cells, SN12C renal cancer cells and MCF7 breast cancer cells	Amen et al., 2013
	H2108, H1299, MRC-5 lung cancer cells; LPS-stimulated RAW264.7 cells	30, 60 µM	Inhibition of cell viability; induction of apoptosis (activation of caspase 3/7; repression of AP-1 and NF-kB-dependent transcription; inhibition of ERK/MAPK pathway); Suppression of (LPS)-induced NO productiwn	Namkoong et al., 2011
	Eca109, KYSE30 esophageal squamous carcinoma cells (ESCC); Eca109 xenograft mouse model	5, 10, 20 µM; 20 mg/kg daily for 20 days	Inhibition of cell proliferation; increase in radio-sensitivity of ESCC; enhanced irradiation-induced DNA damage, apoptosis, G2/M cell cycle arrest; increase in irradiation-induced ROS generation by suppressing Nrf2 and target genes HO-1 and NQO-1; *in vivo* suppression of tumor growth and expression of Ki-67 and PCNA; more profound in combination with irradiation	Zhang et al., 2017
	786-O, RCC4 clear-cell renal cell carcinoma (ccRCC); 786-O xenograft mouse model	2.5, 5, 10 µM; 40, 80 mg/kg daily for 24 days	Suppression of cell growth; induction of apoptosis; inhibition of cell invasion; increased miR-101 expression; repression of RLIP76 expression; inhibition of Akt *in vivo* suppression of tumor growth and pulmonary metastasis	Wang et al., 2017a
	HCT-116, SW480 colorectal cancer (CRC) cells; HCT-116 xenograft mouse model	5, 10 µM; 25, 50 mg/kg daily for 24 days i.p.	Inhibition of cell proliferation; induction of apoptosis; increased DNA double-strand breaks by inhibiting DNA repair *via* RAD51 downregulation; suppression of CRC tumor growth without adverse effects on normal tissues; downregulation of *in situ* levels of Ki-67, Bcl-2 and RAD51; increased cleaved caspase-3 and Bax in tumor tissues	Li et al., 2019
	PC-3 prostate cancer cells	IC_50_ > 30 µM	Inhibition of cell proliferation	Nana et al., 2012
Anticancer activity	A375, SK-MEL-1 melanoma cells; B16-F10 mouse model of lung metastasis	5, 10 µM; 20, 50 mg/kg daily for 24 days (intragastric route)	Inhibition of cell proliferation; impaired metastatic potential by downregulating COX-2 via the miR-124/SPHK 1 axis; decreased number of lung metastases; decreased COX-2 and SPHK1 expression and increased miR-124 expression in metastatic tissues	Gao et al., 2017
	EC9706, KYSE30 ESCC cell lines; KYSE30 xenograft mouse model	10, 20 µM; 50, 100 mg/kg daily for 30 days	Suppression of cell proliferation and tumor growth; Induction of apoptosis by upregulating the miR-370/PIM1 signaling	Han et al., 2016
	CCRF-CEM, CEM/ADR5000 leukemia cells		Strong inhibition of cell proliferation (degree of resistance = 0.62); induction of G0/G1 cell cycle arrest and apoptosis in CCRF-CEM cells through caspase 3/7 activation, mitochondrial membrane potential loss, and ROS production	Kuete et al., 2016
	MDA-MB-231-pcDNA3, MDA-MB-231- BCRP clone 23 breast cancer cells		Moderate inhibition of cell proliferation (degree of resistance = 1.54)	Kuete et al., 2016
	HCT116 (*p*53^+/+^), HCT116 (*p*53^−/−^) colon cancer cells		Moderate inhibition of cell proliferation (degree of resistance = 0.86)	Kuete et al., 2016
	U87MG, U87MG.Δ*EGFR* glioblastoma cells		Moderate inhibition of cell proliferation (degree of resistance = 0.90)	Kuete et al., 2016
	T47D, MDA-MB-231 breast cancer cells	1, 3, 10 µM	Inhibition of hypoxia-induced and iron chelator-induced HIF-1 activation in T47D cells; inhibition of MDA-MB-231 cell migration and chemotaxis	Liu et al., 2009
Antidiabetic activity	α-glucosidase	IC_50_ = 73.3 µM	Inhibition of α-glucosidase activity	Fu et al., 2018
	Protein tyrosine phosphatase-1B (PTP1B)	IC_50_ = 42.0 µM	Inhibition of PTP1B activity	Na et al., 2006
	PTP1B	IC_50_ = 21.2 µM	Inhibition of PTP1B activity	Trinh et al., 2017
	L6 myotubes; PTP1B	1, 10, 25 µM	Stimulation of basal and insulin-treated glucose-uptake in L6 myotubes by increasing AMPK activation, glucose transporters mRNA expression; moderate inhibition of PTP1B (IC_50_ = 37.52 µM)	Lee et al., 2009b
	Acyl-CoA:diacylglycerol acyltransferase (DGAT)	12.5 µg/ml	Inhibition of DGAT activity	Oh et al., 2009
Neuroprotective activity	Monoamine oxidases (MAOs)	IC_50_ = 25.8, 52.6, 16.8 µM, respectively	Inhibition of mixed mouse total brain MAO, MAO-A and MAO-B activity	Han et al., 2005
	SH-SY5Y neuroblastoma cells	IC_50_ > 25 µM	Attenuation of 6-hydroxydopamine-induced neurotoxicity and ROS generation	Kim et al., 2017
Antiplasmodial activity	*Plasmodium falciparum*	IC_50_ = 1.98 µg/ml	Inhibition of parasite proliferation	Tjahjandarie and Tanjung, 2015b
Anti-HIV	HIV-1 protease	IC_50_ = 30.1 µM	Inhibition of HIV-1 protease activity	Lee et al., 2009a

Akt, protein kinase B; BV/TV ratio, bone volume/total volume ratio; cMyc, myelocytomatosis viral oncogene homolog; COX-2, cyclooxygenase-2; DPPH, l,l-diphenyl-2-picrylhydrazyl; ER, estrogen receptor; ERK, extracellular signal-regulated kinase; GREB1, growth regulation by estrogen in breast cancer 1; HIF-1, hypoxia-inducible factor-1; HO-1, heme oxygenase-1; ICAM-1, intercellular adhesion molecule-1; IZ, inhibition zone; JNK, c-Jun N-terminal kinases; LRH-1, liver receptor homologue 1; MAPKs, mitogen-activated protein kinases; MIC, minimum inhibitory concentration; NF-κB, nuclear factor-kappa B; NLRP3, nucleotide-binding domain-like receptor protein 3; NQO-1, NADPH:quinoneoxidoreductase-1; PARP; poly-ADP Ribose polymerase; PCNA; proliferating cell nuclear antigen; PIM1; Pim family kinases 1; RANKL; receptor activator of nuclear factor kappa-B ligand; ROS, reactive oxygen species; SPHK1, sphingosine kinase 1; Tb.N, trabecular number (linear bone density of the trabecular bone); Tb.Sp, trabecular separation (distance between the edges of the trabecular bone); Tb.Th, trabecular thickness.

### Estrogenic and Antiestrogenic Activities

Estrogenic plant-derived products act *via* binding to human estrogen receptors (ERs). AIF was found to be a weak ERα and ERβ binder with conflicting results concerning the preference for ERβ versus ERα (Djiogue et al., 2009; Magne Nde et al., 2012; Mvondo et al., 2012). The authors used the same estrogen receptor competitor assay based on fluorescence polarization in the same laboratory and according to the instructions of the same manufacturer. The discrepancies can probably be ascribed to the purity of compound. The ER competitive ligand binding assay cannot distinguish between estrogenic and antiestrogenic substances and does not provide insight into the ability of a substance to initiate the molecular cascade leading to altered gene expression (Legler et al., 1999). To overcome this disadvantage, reporter gene assays such as the ER-mediated chemically activated luciferase gene expression assay (ER-CALUX) and the yeast estrogen screen (YES) based on stably transfected cell lines are usually applied. In an ER-CALUX assay using human osteosarcoma U2OS cells stably transfected with ERα and transiently transfected with ERβ, AIF stimulated the endogenous ER-estrogen response element (ERE) interaction and, thus, the luciferase reporter gene activity (Djiogue et al., 2009; Magne Nde et al., 2012). However, in a yeast two-hybrid β-galactosidase assay, AIF failed to induce the ligand-dependent interaction of ERα and coactivator TIF2 as determined by the expression of a reporter gene, β-galactosidase (Okamoto et al., 2006). Although ER-CALUX and YES assays rely on the same principle and use the same receptors, the yeast cell wall is usually less permeable to compounds compared to mammalian cell membranes (Legler et al., 1999). This makes the ER-CALUX assay robust, more sensitive and more predictable than the YES assay (Leusch et al., 2010). In MCF-7 cells, AIF upregulated ERα target genes such as proliferating cell nuclear antigen (PCNA), cyclin D1, cyclin E1, cMyc (myelocytomatosis viral oncogene homologue), and liver receptor homologue 1 (LRH-1), and downregulated growth regulation by estrogen in breast cancer 1 (GREB1) (Magne Nde et al., 2012). On the other hand, AIF suppressed estradiol (E2)-induced activity in U2OS-ERβ cells but not in U2OS-ERα cells and ERα yeast two-hybrid systems (Okamoto et al., 2006; Magne Nde et al., 2012). Antagonizing the ERβ-mediated signaling pathway in the presence or absence of E2 is not promising as ERβ is known to counteract the proliferative responses of ERα involved in estrogen-related cancers, osteoporosis, and cardiovascular diseases.

In *in vivo* studies AIF induced estrogen-like effects by increasing uterine wet weight as well as uterine and vaginal epithelial height in ovariectomized Wistar rats (Mvondo et al., 2011, Mvondo et al., 2012). In this model, AIF also reduced the hot flush index by increasing the FSH/LH ratio. It displayed atheroprotective effects by an augmentation of HDL-cholesterol levels, a reduction in the atherogenic index of plasma (Mvondo et al., 2011), and by upregulating the expression of estrogen-sensitive genes associated with bile acid formation (Cyp7a1) (Mvondo et al., 2015). Taken together, the *in vitro* and *in vivo* systems/models used to study estrogenic effects of AIF are quite suitable. The investigations demonstrated that AIF, through activation of ERs and modulation of estrogen-sensitive genes, exhibited estrogenic activities on uterus and vagina and by influencing several factors reduced the atherogenic risk. Nevertheless, further *in vivo* studies are necessary to get deeper insight into its potential.

### Antiosteoporotic Activity

Isoflavonoids are increasingly considered as a promising first-line prophylaxis for osteoporosis in clinical settings (Ma et al., 2008; Lambert and Jeppesen, 2018). The therapeutic strategies globally emphasize the inhibition of “osteoclast-mediated bone resorption” and/or the prevention of the apoptosis of osteoblasts and osteocytes.

Using experimental protocols of postmenopausal or glucocorticoid-induced osteoporosis, AIF exhibited an antiosteoporotic activity both *in vitro* and *in vivo*. A 6-week oral treatment with AIF (10 and 25 mg/kg) prevented ovariectomy-induced osteoporosis in mice by suppressing osteoclast differentiation (Cong et al., 2017). Despite its limits (Egermann et al., 2005; Lelovas et al., 2008), the ovariectomized rat/mouse model is the most widely used animal model in research on postmenopausal osteoporosis. Long-term exposure to glucocorticoids, e.g., in the treatment of chronic autoimmune and pulmonary disorders, cancers of the lymphoid system, as well as in the prevention of transplant rejection (Oakley and Cidlowski, 2013), is the primary cause of the secondary osteoporosis (Tanaka, 2014). In dexamethasone-induced osteoporosis in rats, 20 and 40 mg/kg AIF p.o. prevented bone loss (Wang et al., 2017b). *In vitro*, 5–20 µM AIF abrogated the dexamethasone-induced cytotoxicity and proapoptotic effects on osteoblasts and osteocytes (MC3T3-E1 and MLO-Y4 cells) through the inhibition of ROS production as well as through the activation of nuclear factor erythroid 2-related factor 2 (Nrf2) and AMPK-dependent NAD(P)H oxidase 2 (Nox2) signaling pathways (Wang et al., 2017b; Yin et al., 2018). Osteoporosis remains an important target of research (Hendrickx et al., 2015), and, despite some limits, the two described animal models are appropriate and closer to the human situation than other models. Although further investigation is needed, the available studies showed that AIF, by suppressing osteoclast differentiation or osteoblasts and osteocytes apoptosis, could have beneficial effects on postmenopausal- and glucocorticoid-induced bone damage.

### Antioxidant and Anti-Inflammatory Activities

Through free radical-scavenging and antioxidative effects, antioxidants constitute the first line of defense against the pathogenesis of several diseases (Rani, 2017).

AIF showed radical scavenging activity against l,l-diphenyl-2-picrylhydrazyl (DPPH) radicals (Rahman et al., 2010; Bórquez et al., 2013; Tjahjandarie and Tanjung, 2015a; Akter et al., 2016; Fu et al., 2018). IC_50_ values of 8.30 μg/ml (Rahman et al., 2010), 54.02 μg/ml (Bórquez et al., 2013), 54.80 μM (Fu et al., 2018), and 708.50 μM (Tjahjandarie and Tanjung, 2015a) were determined. In lipopolysaccharide (LPS)-stimulated murine macrophages RAW264.7 and in mice with LPS-stimulated acute lung injury (ALI), 5 and 10 µg/ml AIF significantly increased the production of antioxidative enzymes such as catalase, heme oxygenase-1 (HO-1), glutathione peroxidase, and superoxide dismutase (Li et al., 2018).

There is a long and ever-growing list of *in vitro* antioxidant assays. In the DPPH free radical scavenging assay, quite different IC_50_ values were obtained with AIF probably due to the differences in assay conditions (**Table 3**). Variable DPPH concentrations, incubation times, sample volumes, solvent systems, and pH clearly result in large differences in IC_50_ values (reviewed by Tan and Lim, 2015). To standardize the methodology and ensure comparability between studies or laboratories, a DPPH concentration of 50 µM (for good accuracy), an incubation time of 30 min, and methanol as solvent for less polar samples or buffered methanol for more polar samples have been proposed (Sharma and Bhat, 2009; Mishra et al., 2012). Results expressed in different units additionally impede cross-comparison in many cases. The DPPH assay does not actually measure the antioxidant activity but the reducing capacity of the sample (Benzie and Strain, 1999). Moreover, there is no linear relationship between the antioxidant concentration and the radical scavenging activity. The numerous drawbacks of this assay underline its ineptitude to evaluate the antioxidant capacity. Among the other single electron transfer (SET)-based assays such as the trolox equivalent antioxidant capacity (TEAC), ferric reducing antioxidant power (FRAP), and thiobarbituric acid reactive substances (TBARS), TEAC assay is most popular due to its convenient application which better reflects the antioxidant activity (Floegel et al., 2011). More recently, hydrogen atom transfer (HAT)-based assays like oxygen radical absorbance capacity (ORAC), total radical-trapping antioxidant parameter (TRAP), and crocin-bleaching assays provide better analogies to *in vivo* action (Prior et al., 2005). The main limits of TRAP (which relies only on the lag phase of the kinetic curve for quantitation) and the crocin-bleaching (easily disturbed by compounds absorbing at the monitored wavelength of 450 nm, crocin is not sold as pure compound but as an extract of saffron) assays are probably the reason why the ORAC assay is currently preferred in food and pharmaceutical industries (Huang et al., 2005; Číž et al., 2010; Power et al., 2013). According to Tan and Lim (2015), a mix of SET and HAT-based assays, encompassing several different radical types is recommended to better estimate the overall antioxidant activity of a sample. In summary, the DPPH assay is not appropriate to evaluate the antioxidant activity of a sample. Moreover, the studies recorded in this review did not use the standardized protocols. However, results from the LPS-induced ALI protocol, well known to be associated with the production of ROS and oxidative stress (Su et al., 2014; Yeh et al., 2014), indicate that, *via* an activation of antioxidative enzymes, AIF could be beneficial in the treatment of diseases associated with oxidative stress.

**Table 3:** Assay conditions of the DDPH method in studies recorded in this review.

Studies	DPPH concentration	Solvent for sample (pH)	Sample volume	Incubation time (min)	Standard compound
Bórquez et al. (2013)	400 µM	Methanol (–)		30	Quercetin
Fu et al. (2018)	–	–	–	–	Propyl gallate
Rahman et al. (2010)	20 mg/l	Methanol (–)		20	*Tert*-butyl-1-hydroxytoluene
Tjahjandarie and Tanjung (2015a)	500 µM	Methanol + 0.1 M buffer acetate (pH 5.5)	–	30	Ascorbic acid

–, not indicated.

The overproduction of free radicals is usually associated with excessive or sustained inflammatory reactions (Dandekar et al., 2015). Administered 1 h before LPS challenge, AIF (10 mg/kg i.p.) alleviated LPS-induced lung lesions, pulmonary edema, and hemorrhages by reducing the activity of myeloperoxidase (Li et al., 2018). In this model and in LPS-induced RAW264.7 cells, 5 and 10 µg/ml AIF inhibited the production of proinflammatory mediators including tumor necrosis factor (TNF)-α, interleukin (IL)-6, IL-1b, intercellular adhesion molecule-1 (ICAM-1), and nitric oxide (NO). The mechanisms underlying these activities include the suppression of nuclear factor-kappa B (NF-κB), mitogen-activated protein kinases (MAPKs), the nucleotide-binding domain-like receptor protein 3 (NLRP3) inflammasome, and IL-17 signaling pathways (Li et al., 2018). A similar effect on LPS-induced NO production in RAW264.7 cells was observed by Namkoong et al. (2011) with an IC_50_ value of 15.97 µM. In GOLD docking fitness, AIF displayed a fitness score of 35.42 against cyclooxygenase (COX)-2, an inducible enzyme only expressed after an inflammatory stimulus (Uddin et al., 2014). Four hours after administration, AIF (25 mg/kg i.p.) reduced the carrageenan-induced rat paw edema by 29% in male Wistar rats, whereas the positive control indomethacin (10 mg/kg i.p.) reduced it by 41.67% (Amen et al., 2013). The models used in studies recorded in this review including the carrageenan-induced rat paw edema and the LPS-induced ALI are well-established to study the anti-inflammatory potential of chemicals. Therefore, results from these models provide a strong evidence of the anti-inflammatory potential of AIF.

### Antimicrobial Activity

Over the last decade, the antimicrobial properties of AIF have been evaluated against several drug-resistant and drug-susceptible strains using agar disk diffusion (Kuete et al., 2008), broth microdilution (Kuete et al., 2008; Chukwujekwu et al., 2011; Wang et al., 2014; Ayine-Tora et al., 2016) and macrodilution assays (San-Martín et al., 2015), thin layer chromatography (TLC) bioautography (Akter et al., 2016), and computer-based (virtual) (Sathishkumar and Tharani, 2017) methods.

Tuberculosis (TB) is the leading human infectious-related cause of death (WHO, 2017). Usually, nonpathogenic mycobacterial species such as *Mycobacterium smegmatis* are used as model systems (Altaf et al., 2010; Namouchi et al., 2017). *M. smegmatis* displays an identical susceptibility to that of multidrug-resistant (MDR) clinical isolates of *Mycobacterium tuberculosis* for the two frontline anti-TB drugs isoniazid and rifampicin (Chaturvedi et al., 2007). AIF displayed a minimum inhibitory concentration (MIC) of 19.53 μg/ml and a minimum bactericidal concentration (MBC) of 39.06 μg/ml against *M. smegmatis* MC2 155 (32-fold less active than ciprofloxacin) while showing no activity against *M. smegmatis* ATCC14468 (San-Martín et al., 2015) and *M. tuberculosis* H37Rv (Kuete et al., 2008).

AIF also prevented the growth of Gram-negative (*Enterobacter cloacae*, *Escherichia coli*, *Morganella morganii*, and *Proteus mirabilis*) and Gram-positive (*Staphylococcus aureus* and *Bacillus stearothermophilus*) bacteria with inhibition zones (IZ) of 15.5–18.5 mm. Only the effects against *E. coli* (IZ = 7.0 mm) were weaker. MIC values of 39.06 μg/ml (against *E. cloacae*, *M. morganii*, *S. aureus*, and *B. stearothermophilus*) and an MBC of 78.12 μg/ml (only against *M. morganii*) were determined. In this series, AIF was less active than the reference gentamycin (IZ = 23.8–31.7 mm; MIC = 2.44–9.76 μg/ml) (Kuete et al., 2008). In a study by Chukwujekwu et al. (2011), AIF displayed MICs of 3.9 μg/ml (against *S. aureus*, *E. coli*, and *Klebsiella pneumoniae*) and 7.8 μg/ml (against *Bacillus subtilis*), while those of the reference neomycin ranged between 0.78 and 1.6 μg/ml. Weak inhibitory activity (MIC ≥ 64 μg/ml) was observed against drug-resistant (SA1199B, RN4220, EMRSA-15, XU212, and EMRSA-16) and wild-type strains of *S. aureus* (Wang et al., 2014). Using a TLC bioautography assay, Akter et al. (2016) reported minimum inhibitory quantities of 15 μg for methicillin-sensitive *S. aureus* and 30 μg for a methicillin-resistant and an isolated multidrug resistant strain of *S. aureus*. By contrast, AIF did not show antibacterial activity against the clinical isolates of *S. aureus*, *M. morganii*, *E. coli*, and *Klebsiella granulomatis* in a study by San-Martín et al. (2015). This might be explained by differences in the methods and microbial strains.

*Chlamydia trachomatis* is the most common infectious cause of trachoma. By significantly interacting (G.score of –2.5 kcal/mol) with the active site residue GLU-626(O-H) of contact-dependent secretion D (CdsD) protein *in silico*, AIF might disrupt the assembly of the type III secretion system (T3SS) involved in differentiation, replication, and dissemination *C. trachomatis* (Sathishkumar and Tharani, 2017).

AIF was fungistatic against wild (MIC = 0.25 μg/ml) and ATCC18804 (MIC = 0.50 μg/ml) strains of *Candida albicans* (Ayine-Tora et al., 2016). At the concentration of 50 μg/ml, AIF was not able to inhibit the activity of fatty acid synthase (a potential antifungal target) and the growth of *C. albicans* ATCC90028 and *Cryptococcus neoformans* ATCC90113 (Li et al., 2002). It did not display any activity against *Candida glabrata* (Kuete et al., 2008).

The antibacterial activity of flavonoids is often widely conflicting mainly due to the use of different nonstandardized techniques. To overcome this issue, the Clinical and Laboratory Standards Institute (CLSI) and the European Committee for Antimicrobial susceptibility testing (EUCAST) have approved and published some guidelines over the last two decades (CLSI, 1998; CLSI, 2002; CLSI, 2004; CLSI, 2008; CLSI, 2010a; CLSI, 2010b; CLSI, 2012a; CLSI, 2012b; EUCAST Definitive Document, 2000; EUCAST Discussion Document, 2003). However, despite these guidelines for agar dilution, broth microdilution, and broth macrodilution, results from nonstandardized protocols are still published even in highly reputable journals. Although the disk diffusion technique is easy to apply without specialized equipment, and cheap, the determined IZ value is not related to the antibacterial activity but depends on polarity, concentration, and molecular weight of compounds (Tan and Lim, 2015). Thus, highly polar compounds display a high IZ, and many compounds with the same diffusion rate result in quite different antimicrobial activities. This method is only useful for a simple qualitative screening. It does not allow the quantification of the amount of the antimicrobial agent diffused into the agar, impeding the determination of MICs and MBCs (Ncube et al., 2008; Balouiri et al., 2016). The broth macrodilution or microdilution assays are among the most appropriate methods to determine MIC and MBC values despite the fact that they are unsuitable for highly nonpolar compounds (Tan and Lim, 2015). The reproducibility and the low price due to small amounts of reagents are the main advantages of the microdilution assay over the macrodilution assay. The latter is tedious to perform, requires a lot of manual handling, and is associated with a risk of errors in the preparation of antimicrobial solutions (Jorgensen and Ferraro, 2009). Accordingly, the microdilution method appears to be more accurate. In general, the interpretation of the efficacy depends on the profound knowledge of the model and the used protocol. Nevertheless, stringent endpoint criteria have been set to MIC values of <10 µg/ml or <25 µM for promising plant compounds (Ríos and Recio, 2005; Cos et al., 2006). According to this criterion, AIF could be considered promising only against *S. aureus* ATCC12600, *E. coli* ATCC11775, *K. pneumonia* ATCC13883, *B. subtilis* ATCC6051, and wild and ATCC18804 strains of *C. albicans*. Overall, despite the limits of the used assays/protocols and the discrepancies in results, recorded data suggest the potential of AIF to act as an antimicrobial drug against few microorganisms.

### Anticancer Activity

Different studies reported promising anticancer activities of plant-derived (iso)flavonoids (Magne Nde et al., 2015; Nwodo et al., 2016; Patil and Masand, 2019) including the suppression of proliferation, migration/invasion, tumor angiogenesis and metastasis, and the promotion of apoptosis in various cancers.

In several studies the 3-(4,5-dimethylthiazole-2-yl)-2,5-diphenyltetrazolium bromide (MTT) assay was applied to determine the cytotoxic effects of AIF: The compound exhibited strong cytotoxicity against human oral epidermoid carcinoma KB cells (IC_50_ = 4.13 μg/ml) (Nkengfack et al., 2001) and murine leukemia P-388 (IC_50_ = 4.31 μg/ml) cells (Tjahjandarie and Tanjung, 2015a). IC_50_ values of 19, 34, and 41 μM, respectively, were observed against human leukemia HL-60, K-562, and MOLT-4 cell lines (Kumar et al., 2013). In human lung H2108, H1299, and MRC-5 cancer cell lines, AIF displayed moderate cytotoxicity with IC_50_ values of 33.5, 38.8, and 52.5 µM, respectively (Namkoong et al., 2011). At the concentration of 10 µM, the growth and invasion of the human clear-cell renal carcinoma ccRCC 786-O and Caki1 cells were suppressed by 40 and 50–60%, respectively (Wang et al., 2017a). An IC_50_ value of >25 μM was obtained against human prostate PC-3 (Nana et al., 2012) and neuroblastoma SH-SY5Y (Hong et al., 2018) cancer cells. At the concentration of 10^−5^ M, AIF inhibited the growth of the renal SN12C cancer cells by 32.67% (Amen et al., 2013). AIF (10 μg/ml) displayed a low antiproliferative activity (30–40% inhibition) against the human melanoma A375 and SK-MEL-1 cells after a 24-h incubation and suppressed the migration and invasion of these cell lines (Gao et al., 2017). However, after 48 h of incubation, AIF did not exhibit inhibitory effects against SK-MEL-28 cells in a study by Hu et al. (2017). The degree of cytotoxicity in MTT assay increases with the cell number, the concentration of MTT, and the incubation time (van Tonder et al., 2015). The concentration of MTT was not indicated in the two latter studies, and the cell number (5 × 10^3^ cells/well) was only indicated by Hu et al. (2017). Comparison of the incubation times showed that the higher incubation time (Hu et al., 2017) was associated with lower antiproliferative activity. Although neglected in the vast majority of studies, long incubation times are often associated with the decomposition, metabolism, or precipitation of compounds (Ateba et al., 2018).

Under use of a cell counting kit-8 (CCK-8) assay, AIF exhibited a moderate antiproliferative activity against human esophageal squamous carcinoma (ESCC) Eca109 and KYSE30 cells and at 5 µM enhanced the sensitivity of these cell lines to irradiation (Zhang et al., 2017). In the same model, it also reduced the viability of colorectal HCT-116 and SW480 cancer cells (IC_50_ of 10 and 5 µM, respectively) (Li et al., 2019). The enzyme-based methods including CCK-8 and MTT assays are easy to use, safe, and have a high reproducibility. However, the toxicity of MTT as well as interference of polyphenols with the tetrazolium MTT dye has to be taken into consideration (Wang et al., 2010). A further advantage of the CCK-8 method is its far higher sensitivity (https://www.dojindo.eu.com/Shared/Flyers/Flyer_CCK-8-Rev.pdf)

At 10^−5^ M, AIF inhibited the growth of leukemia CCRF-CEM, MOLT-4, and HL-60 cancer cells by 51.17, 26.15, and 15.49%, respectively. In this study, the type of assay was not specified (Amen et al., 2013).

Induction of apoptosis is a very important property of anticancer drug candidates. In 786-O and Caki1 cells, AIF led to apoptosis by modulating the miR-101/RLIP76 signaling pathway through the inhibition of Akt (Wang et al., 2017a). In addition to the induction of DNA damage and cell cycle arrest (Zhang et al., 2017), AIF induced apoptosis in ESCC cells by upregulating the miR-370/Pim family kinases 1 (PIM1) signaling (Han et al., 2016) and by suppressing the expression of Nrf2, HO-1 and NADPH:quinine oxidoreductase-1 (Zhang et al., 2017). In HL-60 leukemia cells, apoptotic cell death was observed *via* the suppression of NF-κB and the signal transducer and activator of transcription (STAT) signaling pathway (Kumar et al., 2013). AIF induced lung tumor apoptotic cell death by repressing both the ERK/MAPK and NF-κB pathways (Namkoong et al., 2011). In HCT-116 and SW480 cells, it triggered apoptosis by blocking DNA damage repair mediated by the DNA double-strand break repair gene RAD51 (Li et al., 2019) and in CCRF-CEM cells through the loss of MMP and production of ROS (Kuete et al., 2016).

Drug resistance constitutes a major impediment to effective cancer treatment. AIF displayed antiproliferative effects against several MDR cancer cell lines. Strong antiproliferative activities were obtained for both the drug-sensitive CCRF-CEM (IC_50_ = 9.6 μM) and the multidrug-resistant P-glycoprotein-overexpressing subline CEM/ADR5000 (IC_50_ = 5.91 μM) cells (Kuete et al., 2016). In other drug-sensitive cell lines [breast MDA-MB-231-pcDNA3, colon HCT116 (p53^+/+^), glioblastoma U87MG] and their MDR counterparts [MDA-MB-231-*BCRP* clone 23, HCT116 (p53^−/−^) and U87MG.*ΔEGFR*], AIF displayed moderate effects with IC_50_ values of 42.4–46.7 and 36.4–65.6 μM, respectively. In comparison to normal AML12 hepatocytes, a selective index >3.13 was observed towards HepG2 liver cancer cells (Kuete et al., 2016).

The role of increased activity of hypoxia-inducible factor-1 (HIF-1), especially HIF-1α is well known in cancer progression (Massoud and Li, 2015; Schito and Semenza, 2016). Hypoxic cancer cells seem to be resistant to radiation and chemotherapy (Rohwer and Cramer, 2011; Zhang et al., 2015). Therefore, targeting HIF-1 is an important approach for cancer prevention and treatment. AIF suppressed both hypoxia-induced and iron chelator-induced HIF-1 activation in T47D human breast cancer cells as well as MDA-MB-231 cell migration (Liu et al., 2009).

The antiproliferative or cytotoxic activity associated with apoptosis in malignant cells is a highly important target in the screening of anticancer drugs. Given the severe adverse reactions in normal tissues by tumoricidal doses of chemotherapeutic agents, the cytotoxic activity of drug candidates should also be evaluated against normal cells. In addition to a strong antiproliferative activity (IC_50_ <4 µg/ml or <10 µM for a pure compound after 48–72 h incubation) (Boik, 2001), a high selectivity (selectivity index ≥3) towards malignant cells is needed. The inclusion of positive controls in respective studies of natural compounds is indispensable in good experimental practice (Ateba et al., 2018). Among the 13 *in vitro* studies recorded in this review, only one investigated the effects against normal cells (Kumar et al., 2013) and four used a positive control (Nana et al., 2012; Tjahjandarie and Tanjng, 2015a; Hu et al., 2017; Hong et al., 2018). In addition to this deficit, human tumor cell lines as the workhorse of cancer research are cultured since decades and do not adequately mirror (different tumor environment) the biology of human tumors (Ben-David et al., 2017). Therefore, *in vivo* models with better and more clinically predictive power of human cancers are an imperative.

*In vivo*, AIF has been tested in various xenograft mouse models. After 30 consecutive days of treatment, the compound reduced tumor growth in KYSE30 (50 and 100 mg/kg/day) and Eca109 (20 mg/kg/day) xenograft mouse models (Han et al., 2016; Zhang et al., 2017). It also suppressed the tumor growth in an HCT-116 xenograft mouse model after 24 days treatment (25 and 50 mg/kg/day AIF i.p.) (Li et al., 2019). In a B16-F10 mouse lung model of metastasis, 24-day intragastrical administration of 20 and 50 mg/kg/day AIF decreased the number of metastatic pulmonary nodules. The reduction in COX-2 through modulating miR-124/SPHK1 axis was the underlying mechanism involved (Gao et al., 2017). The dose of 40 and 80 mg/kg/day for 24 days suppressed the growth and pulmonary metastatic nodules in a 786-O xenograft mouse model by modulating miR-101/RLIP76 signaling (Wang et al., 2017a). In a study by Zhang et al. (2017), combination of AIF (20 mg/kg/day for 30 days) with irradiation induced a more profound tumor regression than single treatments. All these *in vivo* activities occurred without affecting the body weight of the mice. Despite the drawbacks of the majority of the current cell-line-derived or patient-derived mouse xenograft models reviewed by Landgraf et al. (2018), they have become a prominent cancer model system over decades. For research in pharmaceutical industry, the accurate description of materials and methodology is indispensable to assure that experiments can be accurately replicated. However, the route of administration of AIF, an extremely important parameter, is not mentioned in the studies by Zhang et al. (2017) and Wang et al. (2017a), published in “high-impact” journals. As a different route of administration leads to different results, this underlines the importance of an accurate review of such papers. Nevertheless, all data reported in this section demonstrate that AIF could have a potential to suppress some tumor growth *in vivo*.

### Antidiabetic Activity

Adequate glycemic control remains the main foundation of managing diabetes mellitus (DM) (Chaudhury et al., 2017). Retarding the release of D-glucose from dietary carbohydrates and delaying its absorption through the inhibition of α-glucosidase is an attractive therapeutic target for the treatment of DM, obesity, and other related complications (van de Laar et al., 2005). *In vitro*, AIF exhibited a moderate α-glucosidase inhibitory activity with an IC_50_value of 73.3 ± 12.9 μM (Fu et al., 2018).

Protein tyrosine phosphatase 1B (PTP1B) is a negative key regulator of insulin signaling pathways that leads to insulin resistance. Thus, it is a promising molecular-level therapeutic target in the management of type 2 DM and obesity (Wang et al., 2015). In a study by Na et al. (2006), AIF exhibited *in vitro* PTP1B inhibitory activity with an IC_50_ value of 42 μM as compared to the positive controls RK-682 (IC_50_ = 4.5 ± 0.5 μM) and ursolic acid (IC_50_ = 3.6 ± 0.2 μM) (Trinh et al., 2017). By increasing the AMPK activation and the expression of glucose transporters’ (GLUT-4 and -1) mRNA as well as by inhibiting the PTP1B activity (IC_50_ = 37.52 µM vs. ursolic acid—5.13 µM), AIF significantly stimulated the glucose uptake in L6 myotubes (Lee et al., 2009b). These differences in IC_50_ values can be explained by the application of different experimental conditions. Using a nonkinetic method to estimate the amount of produced *p*-nitrophenol at 405 nm, Na et al. (2006) added 10 M NaOH to stop the reaction, while in the study of Trinh et al. (2017), the release rate of *p*-nitrophenol (kinetic method) was determined by measuring the absorbance at 405 nm every 30 s for 10 min. Moreover, these studies used different concentrations of PTP1B and the substrate *p*-nitrophenyl phosphate.

Acyl-CoA:diacylglycerol acyltransferase (DGAT) is a key enzyme in the synthesis of triglycerides, the imbalance of which usually leads to insulin resistance and type 2 DM. At the concentration of 12.5 μg/ml, AIF induced 23% inhibition of the activity of this enzyme, while the positive control displayed an IC_50_ value of 4.8 µg/ml (Oh et al., 2009).

Overall, these preliminary results suggest that AIF could exhibit a potential for the treatment of type 2 DM by retarding the glucose absorption from small intestine, by increasing the insulin sensitivity and the glucose transport into cells, and by improving triglycerides’ profile. But most important, this hypothesis has to be confirmed by respective meaningful *in vivo* models.

### Neuroprotective Activity

Elevation of the activity of brain monoamine oxidases (MAOs), especially MAO-B, contributes to chronic neurodegeneration and brain atrophy (Naoi et al., 2018; Tong et al., 2017). AIF inhibited the mixed type of mouse total brain MAO with an IC_50_ value of 25.8 μM. Its activity on MAO-B (IC_50_ = 16.8 μM) was 3.1-fold higher than that on MAO-A (Han et al., 2005). Globally, AIF was more active than the positive control amitriptyline on mixed MAO, MAO-A, and MAO-B. By destroying dopaminergic and noradrenergic neurons in the brain through excessive production of ROS such as superoxide radicals, the neurotoxin 6-hydroxydopamine (6-OHDA) induces neuronal cell death and Parkinson’s disease in rats (Heikkila et al., 1989; Perese et al., 1989; Schober, 2004). At noncytotoxic concentrations, AIF attenuated (IC_50_ > 25 μM) the 6-OHDA-induced neurotoxicity and ROS generation in SH-SY5Y cells (Kim et al., 2017).

The relatively high MAO inhibitory activity of AIF compared to amitriptyline and its capacity to protect against 6-OHDA-induced neurotoxicity justifies further in-depth investigations of AIF for its potential in neurodegenerative diseases such as Parkinson’s and Alzheimer’s.

### Other Activities

With 216 million cases and 445,000 deaths in 2016, malaria remains a major cause of death worldwide, especially in Africa (http://www.who.int/malaria/en/). AIF has shown strong antiplasmodial properties against *Plasmodium falciparum* with an IC_50_ value of 1.98 µg/ml as compared with the positive control chloroquine (IC_50_ = 1.02 µg/ml) (Tjahjandarie and Tanjung, 2015b).

HIV-1 protease and reverse transcriptase are the most important targets in the search for anti-HIV agents. *In vitro*, AIF showed a low inhibitory activity against HIV-1 protease with an IC_50_ value of 30.1 μM (Lee et al., 2009a).

## Structure-Activity Relationship

Numerous prenyl- (Hu et al., 2017), O-methyl- (Waffo et al., 2000; Han et al., 2005; Liu et al., 2009; Lim et al., 2012; Ndemangou et al., 2013; Ayine-Tora et al., 2016; Ocloo et al., 2017; Fu et al., 2018), and/or O-acetyl (Bórquez et al., 2013; Ayine-Tora et al., 2016) derivatives of AIF have been detected in various plants and studied for the impact on the biological activities. From studies comparing both the activities of AIF and those of one or more of its derivatives (**Table 4**), it can be deduced that:

- The replacement of C4′-OH and/or C5-OH by −OMe or O-acetyl reduces the antifungal activity against *C. albicans* (Ayine-Tora et al., 2016).
- The 4′-O-methylated form of AIF promoted the inhibition of HIF-1 activation in T47D cells, the MDA-MB-231 cell migration (Liu et al., 2009), and the inhibition of urease (Ndemangou et al., 2013) and MAO-A (Han et al., 2005) activities, while no significant changes on the influence on MAO-B and α-glucosidase activities were observed (Han et al., 2005; Fu et al., 2018).
- In AIF, initially inactive, the introduction of a prenyl group at the C-8 position to obtain scandenolone or warangalone significantly increased the growth inhibitory activity (IC_50_ < 5 μM) towards human melanoma SK-MEL-28 cells (Hu et al., 2017).
- The [1, 2- *b*:5, 4- *b*’] dipyran derivative derrone showed antiproliferative activity in human leukemia U937 cells in a similar magnitude like AIF (Matsuda et al., 2007). The same refers to the inhibition of PTP1 (IC_50_ = 12.6 µM for derrone and 21.6 µM for alpinumisoflavone) (Trinh et al., 2017). In contrast, derrone was moderately inhibiting phospholipase Cγ1 activity and the formation of inositol phosphates in phospholipase Cγ1-overexpressing NIH3T3 fibroblasts, whereas AIF remained without effect (Oh et al., 2005).

**Table 4:** Impact of different substitutions on the activity of alpinumisoflavone.

Substituent	Impact on the activity	Experimental model	References
**4′-O-methyl**	↑ Inhibition of hypoxia-inducible factor-1 (HIF-1) activation	Human breast tumor T47D cells	Liu et al., 2009
	↑ Inhibition of tumor cell migration and chemotaxis	MDA-MB-231 cells	Liu et al., 2009
	↓ Antiradical activity	DPPH assay	Fu et al., 2018
	↔Inhibition of α-glucosidase activity	α-Glucosidase enzyme model	Fu et al., 2018
	↓ Antifungal activity	*Candida albicans* (wild and ATCC18804 strains)	Ayine-Tora et al., 2016
	↑ Monoamine oxidase-A (MAO-A) activity ↔ MAO-B activity	Mitochondrial fraction from mouse brain	Han et al., 2005
	↑ Inhibition of urease activity	*Helicobacter pylori* urease enzyme assay	Ndemangou et al., 2013
**O,O-dimethyl**	↓ Antifungal activity	*Candida albicans* (wild and ATCC18804 strains)	Ayine-Tora et al., 2016
**5-O-acetyl- and 4′-O-methyl**	↓ Antifungal activity	*Candida albicans* (wild and ATCC18804 strains)	Ayine-Tora et al., 2016
**4´-O-acetyl**	↓ Antiradical activity	DPPH assay	Bórquez et al., 2013
**8-prenyl**	↑ Antiproliferative activity	Human melanoma SK-MEL-28 cells	Hu et al., 2017

↑; increase, ↓; decrease, ↔; not different.

The differences in the activities of some compounds closely related to AIF, preclinically tested in comparable assays but not directly compared with AIF, are difficult to interpret due to the limitations as discussed above. Nevertheless, we include those results, which were obtained in the most similar experimental setups:

- The 2′-OH derivative parvisoflavone B showed a stronger α-glucosidase inhibition (IC_50_ = 12.2 µM; Dendup et al., 2014) than AIF (IC_50_ = 73.3 µM; Fu et al., 2018). Nevertheless, due to the lack of a positive control in the study with AIF, a comparison of the results remains questionable. Parvisoflavone B resulted also in a better cytotoxic effect against MDA-MB-231 breast cancer cells (EC_50_ = 16.9 µM) (Nyandoro et al., 2017). The weak antimycobacterial effect (MIC = 90.9 µM) of parvisoflavone B against *M. tuberculosis* H37rV) (Nyandoro et al., 2017) differed from the inactive AIF (Kuete et al., 2008).
- Cudraisoflavon M—carrying an additional 2,3-dihydroxy-prenyl group at C-8 of AIF—did not show any activity against 6-OHDA-induced cell death in SH-SY5Y cells (Hiep et al., 2017), whereas cudraisoflavon H with a prenyl group at C-8 and an additional hydroxy group at C-2″ of AIF resulted in an IC_50_ value of 4.5 µM (Hiep et al., 2015).
- The comparison of the antimicrobial effects of derrone with AIF is extremely difficult due to the differences in the experimental setup as discussed above. Nevertheless, the activity of derrone against *E. coli*, *S. aureus*, and *C. albicans* seems lower than the one of AIF (Edziri et al., 2012). The antiproliferative potential of derrone and AIF differ in dependence of the cell line: In SW480 cells, AIF with an IC_50_ value of 5 µM inhibits the proliferation (Li et al., 2019), whereas derrone remains inactive (Li et al., 2017). In MCF-7 cells, AIF was inactive (Sudanich et al., 2017) and derrone at 10 µM inhibited this cell line by 13.6% (Li et al., 2017). HepG2 cells seem to be similarly sensitive to the two compounds [AIF—IC_50_ 37.99 µM (Kuete et al., 2016); derrone—23.7% inhibition at 10 µM (Li et al., 2017)].
- 2′-Hydroxyerythrin A with the OH group from C-5 shifted to C-2′ showed good activity against several Gram-positive and Gram-negative bacteria (Wang et al., 2018). The magnitude of the DPPH radical scavenging effect was in the same range (Wang et al., 2018) as in some studies with AIF (Rahman et al., 2010; Bórquez et al., 2013; Fu et al., 2018).

The infrequence of studies and the low number of different substitution patterns and of investigated activities are the main drawback in the deduction of structure–activity relationships (SAR) of AIF and its derivatives. Continued efforts are needed to further synthesize or isolate new derivatives of AIF to expand SAR. Nevertheless, published data indicate that the C4′-O-methylation and the C8-prenylation increase the activity of AIF in cancer and neurodegenerative conditions. This is in accordance with Bernini et al. (2011) who indicated that O-methylation of flavonoids ensures a superior anticancer activity as compared with the corresponding hydroxylated derivatives, since such compounds are more resistant to hepatic metabolism and show higher intestinal absorption. In addition, Walle et al. (2007) suggested that O-methylation enhances the stability of flavonoids to metabolic degradation and increases their bioavailability as well as a higher tissue distribution as compared to unmethylated forms.

## Point of View and Future Perspective

Prenylated (iso)flavonoids are attracting more and more attention due to a series of promising biological activities ascribed to their increased lipophilicity and a strong affinity to biological membranes as compared to the respective unprenylated compounds (Botta et al., 2005; Botta et al., 2009; Chen et al., 2014; Sherif et al., 2015; Mukai, 2018). In this context, numerous pharmacological investigations of alpinumisoflavone, extracted from various medicinal plants, were carried out over the last decades. Data recorded in this review evidence a wide array of activities such as antiosteoporotic, antioxidant, anti-inflammatory, antimicrobial, anticancer, estrogenic and antiestrogenic, antidiabetic, and neuroprotective. Discrepancies between results were usually attributed to the purity of the tested compound, the experimental setup, the operator’s experience, or other experimental parameters (Ateba et al., 2018). Many of related pathologies or conditions such as antimicrobial resistance, cancer, diabetes mellitus, and neurodegenerative diseases are becoming pivotal concerns for public health over the world. However, although AIF might be considered a promising preventive and/or therapeutic agent for such ailments, these investigations are only at the beginning. Using suitable and well-designed standardized models or assays, further and thorough studies related to the above mentioned or other pathologies/conditions are needed to confirm this potential. *In vitro* evaluation is an important primary screen and due to its rapidity common practice in many research laboratories. Nevertheless, many *in vitro* studies are not necessarily optimal due to poor standardization, redundancy, and/or outdated methodology (Tan and Lim, 2015). Clearly, compounds exhibiting promising activity require further studies to validate or confirm their therapeutic potential (Kenny et al., 2015). Accordingly, the correlation with *in vivo* data using appropriate models is an indispensable prerequisite.

The analysis of structure–activity relationships provides information on the preferential conformation to maintain high activities. Studies of AIF until now revealed that the free –OH groups at C-4′ and C-5 are important for the fungicidal activity towards *C. albicans* (Ayine-Tora et al., 2016). 4′-O-Methylation and the presence of a prenyl group at C-8 enhanced anticancer activities (Liu et al., 2009; Hu et al., 2017). Studies with diversified substituents would be ideal for the investigation of SARs. They might allow the identification of important structures with reduced toxicity and increased therapeutic efficacy that can guide the design of novel leads or drug candidates. However, such studies on AIF and its derivatives are scarce until now, and this underlines the necessity of further well-performed investigations.

Besides the efficacy, extensive safety and pharmacokinetic data are required for potential drug candidates as an important aspect in the drug development process. However, till today, no study dealing with the toxicity or pharmacokinetics of AIF has been reported.

## Conclusion

**Figure 2:**
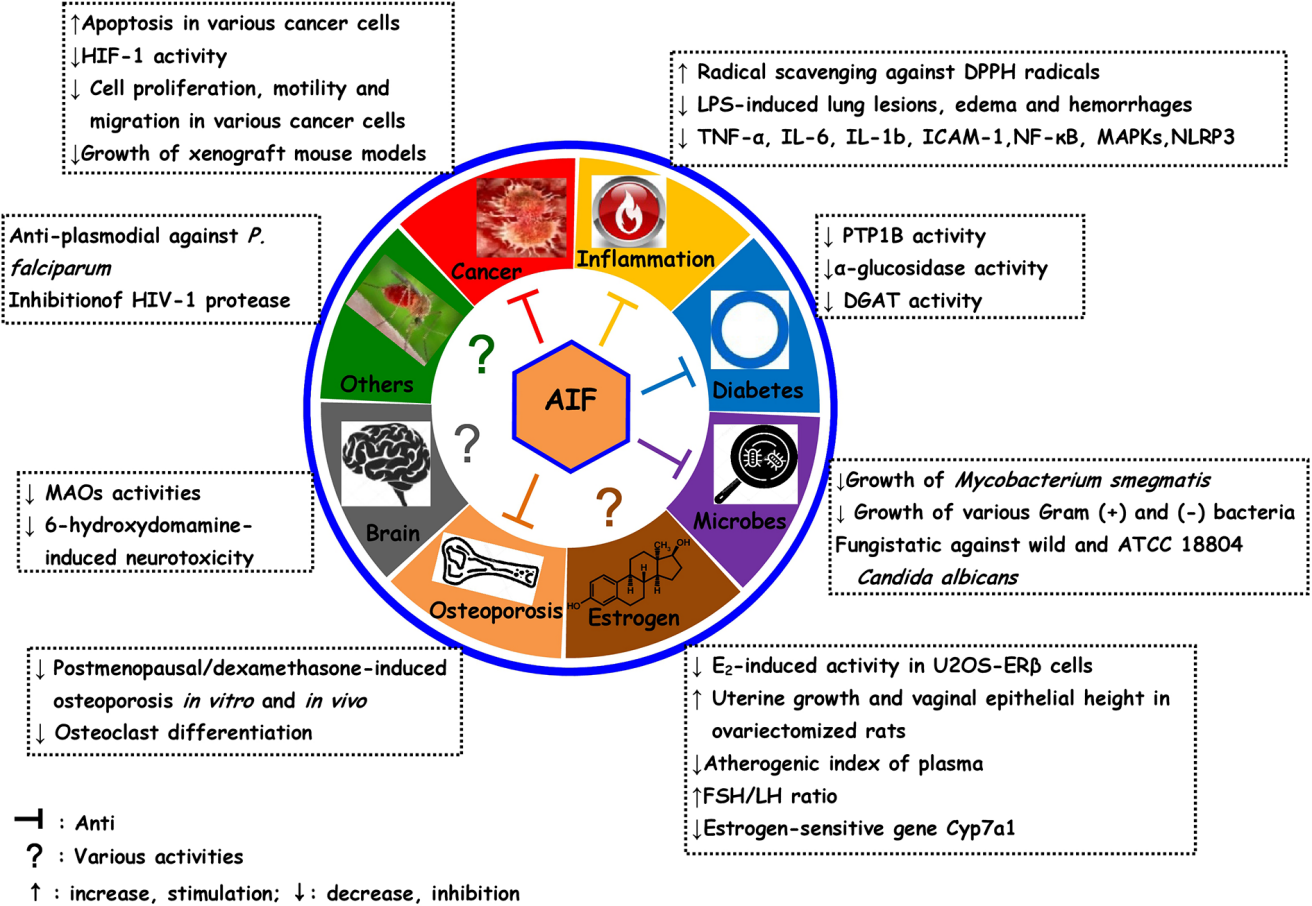
Overview over pharmacological activities of alpinumisoflavone and some of its derivatives.

This review evidences that AIF is a versatile compound with a wide array of possible health benefits. We summarize the current preclinical evidence of the antiosteoporotic, antioxidant and anti-inflammatory, antimicrobial, anticancer, estrogenic and antiestrogenic, antidiabetic, and neuroprotective activities (**Figure 2**). However, more persuasive and scientific evidence and detailed mechanistic studies are urgently needed for a therapeutic exploitation of AIF. Moreover, SAR of AIF and its derivatives indicates that 4′-O-methyl-AIF appears to be more promising than AIF, and these indications need to be investigated in-depth.

## Author Contributions

SA obtained literatures, wrote the first draft, and edited the manuscript; MM obtained literatures and wrote sections of the manuscript. SD, SZ, and DN gave ideas and critically reviewed the manuscript. LK gave ideas, critically reviewed and edited the manuscript. All authors read and approved the manuscript.

## Conflict of Interest Statement

The authors declare that the research was conducted in the absence of any commercial or financial relationships that could be construed as a potential conflict of interest.

## References

[B1] Akter K. Barnes E. C. Loa-Kum-Cheung W. L. Yin P. Kichu M. Brophy J. J. (2016). Antimicrobial and antioxidant activity and chemical characterization of *Erythrina stricta* Roxb. (Fabaceae). J. Ethnopharmacol. 185, 171–181. 10.1016/j.jep.2016.03.011 26969405

[B2] Altaf M. Miller C. H. Bellows D. S. O’Toole R. (2010). Evaluation of the *Mycobacterium smegmatis* and BCG models for the discovery of *Mycobacterium tuberculosis* inhibitors. Tuberculosis 90, 333–337. 10.1016/j.tube.2010.09.002 20933470

[B3] Amen Y. M. Marzouk A. M. Zaghloul M. G. Afifi M. S. (2013). Bioactive compounds from *Tipuana tipu* growing in Egypt. J. Am. Sci. 9 (10), 334–339.

[B4] Ateba S. B. Mvondo M. A. Tchoukouegno Ngueu S. Tchoumtchoua J. Awounfack C. Njamen D. (2018). Natural terpenoids against female breast cancer: a 5-year recent research. Curr. Med. Chem. 25, 3162–3213. 10.2174/0929867325666180214110932 29446727

[B5] Ayine-Tora D. M. Kingsford-Adaboh R. Asomaning W. A. Harrison J. J. E. K. Mills-Robertson F. C. Bukari Y. (2016). Coumarin antifungal lead compounds from *Millettia thonningii* and their predicted mechanism of action. Molecules 21 (10), 1369. 10.3390/molecules21101369 PMC6274499 27754464

[B6] Balouiri M. Sadiki M. Ibnsouda S. K. (2016). Methods for *in vitro* evaluating antimicrobial activity: a review. J. Pharm. Anal. 6, 71–79. 10.1016/j.jpha.2015.11.005 29403965 PMC5762448

[B7] Ben-David U. Ha G. Tseng Y.-Y. Greenwald N. F. Oh C. Shih J. (2017). Patient-derived xenografts undergo mouse-specific tumor evolution. Nature Genetics 49, 1567–1578. 10.1038/ng.3967 28991255 PMC5659952

[B8] Benzie I. F. F. Strain J. J. (1999). Ferric reducing/antioxidant power assay: direct measure of total antioxidant activity of biological fluids and modified version for simultaneous measurement of total antioxidant power and ascorbic acid concentration. Methods Enzymol. 299, 15–27. 10.1016/S0076-6879(99)99005-5 9916193

[B9] Bernini R. Crisante F. Ginnasi M. C. (2011). A convenient and safe O-methylation of flavonoids with dimethyl carbonate (DMC). Molecules 16, 1418–1425. 10.3390/molecules16021418 21307820 PMC6259620

[B10] Boik J. (2001). Natural compounds in cancer therapy. 1st edition. Minnesota: Oregon Medical Press.

[B11] Bórquez J. Kennelly E. J. Simirgiotis M. J. (2013). Activity guided isolation of isoflavones and hyphenated HPLC-PDA-ESI-ToF-MS metabolome profiling of *Azorella madreporica* Clos. from northern Chile. Food Res. Int. 52, 288–297. 10.1016/j.foodres.2013.02.055

[B12] Botta B. Menendez P. Zappia G. de Lima R. A. Torge R. Monache G. D. (2009). Prenylated isoflavonoids: botanical distribution, structures, biological activities and biotechnological studies. An update (1995–2006). Curr. Med. Chem. 16, 3414–3468. 10.2174/092986709789057662 19548871

[B13] Botta B. Vitali A. Menendez P. Misiti D. Monache G. D. (2005). Prenylated flavonoids: pharmacology and biotechnology. Curr. Med. Chem. 12, 713–739. 10.2174/0929867053202241 15790308

[B14] Chaturvedi V. Dwivedi N. Tripathi R. P. Sinha S. (2007). Evaluation of *Mycobacterium smegmatis* as a possible surrogate screen for selecting molecules active against multi-drug resistant *Mycobacterium tuberculosis*. J. Gen. Appl. Microbiol. 53, 333–337. 10.2323/jgam.53.333 18187888

[B15] Chaudhury A. Duvoor C. Reddy Dendi V. S. Kraleti S. Chada A. Ravilla R. (2017). Clinical review of antidiabetic drugs: implications for type 2 diabetes mellitus management. Front. Endocrinol. 8, 6. 10.3389/fendo.2017.00006 PMC5256065 28167928

[B16] Chen L. W. Cheng M. J. Peng C. F. Chen I. S. (2010). Secondary metabolites and antimycobacterial activities from the roots of *Ficus nervosa*. Chem. Biodivers. 7, 1814–1821. 10.1002/cbdv.200900227 20658670

[B17] Chen X. Mukwaya E. Wong M. S. Zhang Y. (2014). A systematic review on biological activities of prenylated flavonoids. Pharm. Biol. 52, 655–660. 10.3109/13880209.2013.853809 24256182

[B18] Chukwujekwu J. C. Van Heerden F. R. Van Staden J. (2011). Antibacterial activity of flavonoids from the stem bark of *Erythrina caffra* Thunb. Phytother. Res. 25, 46–48. 10.1002/ptr.3159 20623615

[B19] Číž M. Čížová H. Denev P. Kratchanova M. Slavov A. Lojek A. (2010). Different methods for control and comparison of the antioxidant properties of vegetables. Food Control 21 (4), 518–523. 10.1016/j.foodcont.2009.07.017

[B20] CLSI (2004). Method for antifungal disk diffusion susceptibility testing of yeasts, approved guideline. CLSI document M44-A. Wayne, Pennsylvania 19087-1898, USA: Clincal and Laboratory Standards Institute, 940 West Valley Road, Suite 1400.

[B21] CLSI (2010a). Method for antifungal disk diffusion susceptibility testing of non-dermatophyte filamentous fungi, approved guideline, CLSI document M51-A. Wayne, Pennsylvania 19087, USA: Clinical and Laboratory Standards Institute, 950 West Valley Road, Suite 2500.

[B22] CLSI (2010b). Methods for antimicrobial dilution and disk susceptibility of infrequently isolated or fastidious bacteria, approved guideline, 2nd. ed., CLSI document M45-A2. Wayne, Pennsylvania 19087, USA: Clinical and Laboratory Standards Institute, 950 West Valley Road, Suite 2500.

[B23] CLSI (1998). Methods for determining bactericidal activity of antimicrobial agents, approved guideline, CLSI document M26-A. Wayne, Pennsylvania 19087, USA: Clinical and Laboratory Standards Institute, 950 West Valley Road Suite 2500.

[B24] CLSI (2012b). Methods for dilution antimicrobial susceptibility tests for bacteria that grow aerobically, approved standard, 9th ed., CLSI document M07-A9. Wayne, Pennsylvania 19087, USA: Clinical and Laboratory Standards Institute, 950 West Valley Road, Suite 2500.

[B25] CLSI (2012a). Performance standards for antimicrobial disk susceptibility tests, approved standard, 7th ed., CLSI document M02-A11. Wayne, Pennsylvania 19087, USA: Clinical and Laboratory Standards Institute, 950 West Valley Road, Suite 2500.

[B26] CLSI (2002). Reference method for broth dilution antifungal susceptibility testing of yeasts, approved standard, 2nd ed., NCCLS document M27-A2. Wayne, Pennsylvania 19087-1898, USA: Clinical and Laboratory Standards Institute, 940 West Valley Road, Suite 1400.

[B27] CLSI (2008). Reference method for broth dilution antifungal susceptibility testing filamentous fungi, approved standard, 2nd ed., CLSI document M38-A2. Wayne, Pennsylvania 19087, USA: Clinical and Laboratory Standards Institute, 950 West Valley Road, Suite 2500.

[B28] Cong W. Zhou C. Yin J. (2017). Alpinumisoflavone inhibits osteoclast differentiation and exerts anti-osteoporotic effect in ovariectomized mice. Biomed. Pharmacother. 93, 344–351. 10.1016/j.biopha.2017.06.059 28651235

[B29] Cos P. Vlietinck A. J. Berghe D. V. Maes L. (2006). Anti-infective potential of natural products: how to develop a stronger *in vitro*‘proof-of-concept’. J. Ethnopharmacol. 106, 290–302. 10.1016/j.jep.2006.04.003 16698208

[B30] Dai J. Shen D. Yoshida W. Y. Parrish S. M. Williams P. G. (2012). Isoflavonoids from *Ficus benjamina* and their inhibitory activity on BACE1. Planta Med. 78, 1357–1362. 10.1055/s-0032-1315001 22763739 PMC4696551

[B31] Dandekar A. Mendez R. Zhang K., (2015). “Cross talk between ER Stress, oxidative stress, and inflammation in health and disease,” in Stress responses. Methods in molecular biology, vol. 1292 Ed. Oslowski C. (New York, NY: Humana Press). 10.1007/978-1-4939-2522-3_15 25804758

[B32] Dendup T. Prachyawarakorn V. Pansanit A. Mahidol C. Ruchirawat S. Kittakoop P. (2014). α-Glucosidase inhibitory activities of isoflavanones, isoflavones, and pterocarpans from *Mucuna pruriens*. Planta Med. 80, 604–608. 10.1055/s-0034-1368427 24782227

[B33] Djiogue S. Halabalaki M. Alexi X. Njamen D. Fomum Z. T. Alexis M. N. (2009). Isoflavonoids from *Erythrina poeppigiana*: evaluation of their binding affinity for the estrogen receptor. J. Nat. Prod. 72, 1603–1607. 10.1021/np900271m 19705860

[B34] Djiogue S. Njamen D. Halabalaki M. Kretzschmar G. Beyer A. Mbanya J. C. (2010). Estrogenic properties of naturally occurring prenylated isoflavones in U2OS humanosteosarcoma cells: structure–activity relationships. J. Steroid. Biochem. Mol. Biol. 120, 184–191. 10.1016/j.jsbmb.2010.04.014 20420908

[B35] Egermann M. Goldhahn J. Schneider E. (2005). Animal models for fracture treatment in osteoporosis. Osteoporos. Int. 16, S129–S138. 10.1007/s00198-005-1859-7 15750681

[B36] Edziri H. Mastouri M. Mahjoub M. A. Mighri Z. Mahjoub A. Verschaeve L. (2012). Antibacterial, antifungal and cytotoxic activities of two flavonoids from *Retama raetam* flowers. Molecules 17, 7284–7293. 10.3390/molecules17067284 22695233 PMC6268215

[B37] EUCAST Definitive Document, 2000 Terminology relating to methods for the determination of susceptibility of bacteria to antimicrobial agents. Clin. Microbiol. Infec. 6 (9), 503–508. 10.1046/j.1469-0691.2000.00149.x 11168186

[B38] EUCAST Discussion Document, 2003 Determination of minimum inhibitory concentrations (MICs) of antibacterial agents by broth dilution. Clin. Microbiol. Infec. 9 (8), 1–7. 10.1046/j.1469-0691.2003.00790.x 12691538

[B39] Floegel A. Kim D. O. Chung S. J. Koo S. I. Chun O. K. (2011). Comparison of ABTS/DPPH assays to measure antioxidant capacity in popular antioxidant-rich US foods. J. Food Compos. Anal. 24, 1043–1048. 10.1016/j.jfca.2011.01.008

[B40] Fu G. Li W. Huang X. Zhang R. Tian K. Hou S. (2018). Antioxidant and alpha-glucosidase inhibitory activities of isoflavonoids from the rhizomes of *Ficus tikoua*. Bur. Nat. Prod. Res. 32, 399–405. 10.1080/14786419.2017.1312391 28423925

[B41] Gao M. Chang Y. Wang X. Ban C. Zhang F. (2017). Reduction of COX-2 through modulating miR-124/SPHK1 axis contributes to the antimetastatic effect of alpinumisoflavone in melanoma. Am. J. Transl. Res. 9, 986–998. 28386327 PMC5375992

[B42] Guangxi Institute of Chinese Medicine, Pharmaceutical Science (1986). Medicinal plants directory of Guangxi. Guangxi: Guangxi People’s Publishing House, 232.

[B43] Han X. H. Hong S. S. Hwang J. S. Jeong S. H. Hwang J. H. Lee M. H. (2005). Monoamine oxidase inhibitory constituents from the fruits of *Cudrania tricuspidata*. Arch. Pharm. Res. 28, 1324–1327. 10.1007/BF02977895 16392662

[B44] Han Y. Yang X. Zhao N. Peng J. Gao H. Qiu X. (2016). Alpinumisoflavone induces apoptosis in esophageal squamous cell carcinoma by modulating miR-370/PIM1 signaling. Am. J. Cancer Res. 6, 2755–2771. 28042498 PMC5199752

[B45] Heikkila R. E. Sonsalla P. K. Duvoisin R. C. (1989). “Biochemical models of Parkinson’s disease,” in Drugs as tools in neurotransmitter research. Neuromethods, vol. 12 Eds. Boulton A. A. Baker G. B. Juorio A. V. (Totowa, New Jersey: Humana Press).

[B46] Hendrickx G. Boudin E. Van Hul W. (2015). A look behind the scenes: the risk and pathogenesis of primary osteoporosis. Nat. Rev. Rheumatol. 11:462–474. 10.1038/nrrheum.2015.48 25900210

[B47] Hiep N. T. Kwon J. Kim D.-W. Hwang B. Y. Lee H.-J. Mar W. (2015). Isoflavones with neuroprotective activities from fruits of *Cudrania tricuspidata*. Phytochemistry 111, 141–148. 10.1016/j.phytochem.2014.10.021 25487308

[B48] Hiep N. T. Kwon J. Kim D.-W. Hong S. Guo Y. Hwang B. Y. (2017). Neuroprotective constituents from the fruits of *Maclura tricuspidata*. Tetrahedron 73, 2747–2759. 10.1016/j.tet.2017.03.064

[B49] Hong S. Kwon J. Hiep N. T. Sim S. J. Kim N. Kim K. H. (2018). The isoflavones and extracts from *Maclura tricuspidata* fruit protect against neuronal cell death in ischemic injury *via* induction of Nox4-targeting miRNA-25, miRNA-92a, and miRNA-146a. J. Funct. Foods 40, 785–797. 10.1016/j.jff.2017.12.011

[B50] Hu Y. Li Z. Wang L. Deng L. Sun J. Jiang X. (2017). Scandenolone, a natural isoflavone derivative from *Cudrania tricuspidata* fruit, targets EGFR to induce apoptosis and block autophagy flux in human melanoma cells. J. Funct. Foods 37, 229–240. 10.1016/j.jff.2017.07.055

[B51] Huang D. Ou B. Prior R. L. (2005). The chemistry behind antioxidant capacity assays. J. Agric. Food Chem. 53, 1841–1856. 10.1021/jf030723c 15769103

[B52] Ito C. Itoigawa M. Tan H. T. W. Tokuda H. Mou X. Y. Mukainaka T. (2000). Anti-tumor-promoting effects of isoflavonoids on Epstein–Barr virus activation and two-stage mouse skin carcinogenesis. Cancer Lett. 152, 187–192. 10.1016/S0304-3835(00)00331-1 10773411

[B53] Jackson B. Owen P. J. Scheinmann F. (1971). Extractives from poisonous British plants. Part I. The structure of alpinumisoflavone, a new pyranoisoflavone from *Laburnum alpinum*. J. Presl. J. Chem. Soc. C 0, 3389–3392. 10.1039/j39710003389 5166862

[B54] Jorgensen J. H. Ferraro M. J. (2009). Antimicrobial susceptibility testing: a review of general principles and contemporary practices. Clin. Infect. Dis. 49, 1749–1755. 10.1086/647952 19857164

[B55] Kenny C. R. Furey A. Lucey B. (2015). A post-antibiotic era looms: can plant natural product research fill the void? Br. J. Biomed. Sci. 72, 191–200. 10.1080/09674845.2015.11665752 26738402

[B56] Khalid S. A. Farouk A. Geary T. G. Jensen J. B. (1986). Potential antimalarial candidates from African plants: an *in vitro* approach using *Plasmodium falciparum*. J. Ethnopharmacol. 15, 201–209. 10.1016/0378-8741(86)90156-X 3520157

[B57] Kim D. W. Kwon J. Sim S. J. Lee D. Mar W. (2017). Orobol derivatives and extracts from *Cudrania tricuspidata* fruits protect against 6-hydroxydopamine-induced neuronal cell death by enhancing proteasome activity and the ubiquitin/proteasome-dependent degradation of α-synuclein and synphilin-1. J. Funct. Foods 29, 104–114. 10.1016/j.jff.2016.12.017

[B58] Kuete V. Mbaveng A. T. Nono E. C. N. Simo C. C. Zeino M. Nkengfack A. E. (2016). Cytotoxicity of seven naturally occurring phenolic compounds towards multi-factorial drug-resistant cancer cells. Phytomedicine 23, 856–863. 10.1016/j.phymed.2016.04.007 27288921

[B59] Kuete V. Ngameni B. Fotso Simo C. C. Kengap Tankeu R. Tchaleu Ngadjui B. Meyer J. J. M. (2008). Antimicrobial activity of the crude extracts and compounds from *Ficus chlamydocarpa* and *Ficus cordata* (Moraceae). J. Ethnopharmacol. 120, 17–24. 10.1016/j.jep.2008.07.026 18718518

[B60] Kumar S. Pandey A. K. (2013). Chemistry and biological activities of flavonoids: an overview. Sci. World J. 2013, 162750. 10.1155/2013/162750 PMC3891543 24470791

[B61] Kumar S. Pathania A. S. Saxena A. K. Vishwakarma R. A. Ali A. Bhushan S. (2013). The anticancer potential of flavonoids isolated from the stem bark of *Erythrina suberosa* through induction of apoptosis and inhibition of STAT signaling pathway in human leukemia HL-60 cells. Chem-Biol. Interact. 205, 128–137. 10.1016/j.cbi.2013.06.020 23850732

[B62] Lambert M. N. T. Jeppesen P. B. (2018). Isoflavones and bone health in perimenopausal and postmenopausal women. Curr. Opin. Clin. Nutr. 21, 475–480. 10.1097/MCO.0000000000000513 30239339

[B63] Landgraf M. McGovern J. A. Fried P. Hutmacher D. W. (2018). Rational design of mouse models for cancer research. Trends Biotechnol. 36, 242–251. 10.1016/j.tibtech.2017.12.001 29310843

[B64] Lapčík O. (2007). Isoflavonoids in non-leguminous taxa: a rarity or a rule? Phytochemistry 68, 2909–2916. 10.1016/j.phytochem.2007.08.006 17904596

[B65] Lee J. S. Oh W. K. Ahn J. S. Kim Y. H. Mbafor J. T. Wandji J. (2009a). Prenylisoflavonoids from *Erythrina senegalensis* as novel HIV-1 protease inhibitors. Planta Med. 75, 268–270. 10.1055/s-0028-1088395 19097000

[B66] Lee M. S. Kim C. H. Hoang D. M. Kim B. Y. Sohn C. B. Kim M. R. (2009b). Genistein derivatives from *Tetracera scandens* stimulate glucose-uptake in L6 myotubes. Biol. Pharm. Bull. 32, 504–508. 10.1248/bpb.32.504 19252305

[B67] Legler J. van den Brink C. E. Brouwer A. Murk A. J. van der Saag P. T. Vethaak A. D. (1999). Development of a stably transfected estrogen receptor-mediated luciferase reporter gene assay in the human T47D breast cancer cell line. Toxicol. Sci. 48, 55–66. 10.1093/toxsci/48.1.55 10330684

[B68] Lelovas P. P. Xanthos T. T. Thoma S. E. Lyritis G. P. Dontas I. A. (2008). The laboratory rat as an animal model for osteoporosis research. Comparative Med. 58, 424–430. PMC2707131 19004367

[B69] Leusch F. L. De Jager C. Levi Y. Lim R. Puijker L. Sacher F. (2010). Comparison of five *in vitro* bioassays to measure estrogenic activity in environmental waters. Environ. Sci. Technol. 44, 3853–3860. 10.1021/es903899d 20423077

[B70] Li D. Li X. Li G. Meng Y. Jin Y. Shang S. (2019). Alpinumisoflavone causes DNA damage in colorectal cancer cells *via* blocking dna repair mediated by RAD51. Life Sci. 216, 259–270. 10.1016/j.lfs.2018.11.032 30448264

[B71] Li P. Y. Liang Y. C. Sheu M. J. Huang S. S. Chao C. Y. Kuo Y. H. (2018). Alpinumisoflavone attenuates lipopolysaccharide-induced acute lung injury by regulating the effects of anti-oxidation and anti-inflammation both *in vitro* and *in vivo*. RSC Adv. 8, 31515–31528. 10.1039/C8RA04098B PMC9085634 35548248

[B72] Li K. Ji S. Song W. Kuang Y. Lin Y. Tang S. (2017). Glycybridins A–K, bioactive phenolic compounds from *Glycyrrhiza glabra*. J. Nat. Prod. 80, 334–346. 10.1021/acs.jnatprod.6b00783 28140583

[B73] Li X. C. Joshi A. S. ElSohly H. N. Khan S. I. Jacob M. R. Zhang Z. (2002). Fatty acid synthase inhibitors from plants: isolation, structure elucidation, and SAR studies. J. Nat. Prod. 65, 1909–1914. 10.1021/np020289t 12502337

[B74] Lim J. Y. Hwang B. Y. Hwang K. W. Park S. Y. (2012). Methylalpinumisoflavone inhibits lipopolysaccharide-induced inflammation in microglial cells by the NF-kappaB and MAPK signaling pathway. Phytother. Res. 26, 1948–1956. 10.1002/ptr.4810 22899404

[B75] Liu Y. Veena C. K. Brian Morgan J. Mohammed K. A. Jekabsons M. B. Nagle D. G. (2009). Methylalpinumisoflavone inhibits hypoxia-inducible factor-1 (HIF-1) activation by simultaneously targeting multiple pathways. J. Biol. Chem. 284, 5859–5868. 10.1074/jbc.M806744200 19091749 PMC2645834

[B76] Lyddiard J. R. A. Whitfield P. J. Bartl A. (2002). Antischistosomal bioactivity of isoflavonoids from *Millettia thonningii* (Leguminosae). J. Parasitol. 88, 163–170. 10.1645/0022-3395(2002)088[0163:ABOIFM]2.0.CO;2 12053958

[B77] Ma D. F. Qin L. Q. Wang P. Y. Katoh R. (2008). Soy isoflavone intake inhibits bone resorption and stimulates bone formation in menopausal women: meta-analysis of randomized controlled trials. Eur. J. Clin. Nutr. 62, 155–161. 10.1038/sj.ejcn.1602748 17392695

[B78] Magne Nde C. B. Njamen D. Fomum S. T. Wandji J. Simpson E. Clyne C. (2012). *In vitro* estrogenic activity of two major compounds from the stem bark of *Erythrina lysistemon* (Fabaceae). Eur. J. Pharmacol. 674, 87–94. 10.1016/j.ejphar.2011.10.031 22079771

[B79] Magne Nde C. B. Zingue S. Winter E. Creczynski-Pasa T. B. Michel T. Fernandez X. (2015). Flavonoids, breast cancer chemopreventive and/or chemotherapeutic agents. Curr. Med. Chem. 22, 3434–3446. 10.2174/0929867322666150729115321 26219391

[B80] Markovski A. (2016). Dynamics of rooting of storehousebush (*Cudrania tricuspidata* (Carrière.) Bur. Ex Lav.). Bur. ex Lav.) cuttings. J. Mountain Agri. Balkans 19, 134–147.

[B81] Massoud G. N. Li W. (2015). HIF-1α pathway: role, regulation and intervention for cancer therapy. Acta Pharm. Sin. B 5, 378–389. 10.1016/j.apsb.2015.05.007 26579469 PMC4629436

[B82] Matsuda H. Yoshida K. Miyagawa K. Asao Y. Takayama S. Nakashima S. (2007). Rotenoids and flavonoids with anti-invasion of HT1080, anti-proliferation of U937, and differentiation-inducing activityin HL-60 from *Erycibe expansa.* Bioorg. Med. Chem. 15, 1539–1546. 10.1016/j.bmc.2006.09.024 17158054

[B83] Mishra K. Ojha H. Chaudhury N. K. (2012). Estimation of antiradical properties of antioxidants using DPPH assay: a critical review and results. Food Chem. 130, 1036–1043. 10.1016/j.foodchem.2011.07.127

[B84] Mukai R. (2018). Prenylation enhances the biological activity of dietary flavonoids by altering their bioavailability. Biosci. Biotech. Bioch. 82, 207–215. 10.1080/09168451.2017.1415750 29307271

[B85] Mvondo M. A. Njamen D. Tanee Fomum S. Wandji J. (2012). Effects of alpinumisoflavone and abyssinone V-4′-methyl ether derived from *Erythrina lysistemon* (Fabaceae) on the genital tract of ovariectomized female Wistar Rat. Phytother. Res. 26, 1029–1036. 10.1002/ptr.3685 22183714

[B86] Mvondo M. A. Njamen D. Fomum S. T. Wandji J. Vollmer G. (2011). A postmenopause-like model of ovariectomized Wistar rats to identify active principles of *Erythrina lysistemon* (Fabaceae). Fitoterapia 82, 939–949. 10.1016/j.fitote.2011.05.009 21635940

[B87] Mvondo M. A. Njamen D. Kretzschmar G. Bader M. I. Fomum S. T. Wandji J. (2015). Alpinumisoflavone and abyssinone V 4′-methylether derived from *Erythrina lysistemon* (Fabaceae) promote HDL-cholesterol synthesis and prevent cholesterol gallstone formation in ovariectomized rats. J. Pharm. Pharmacol. 67, 990–996. 10.1111/jphp.12386 25683903

[B88] Na M. K. Jang J. P. Njamen D. Mbafor J. T. Fomum Z. T. Kim B. Y. (2006). Protein tyrosine phosphatase-1B inhibitory activity of isoprenylated flavonoids isolated from *Erythrina mildbraedii*. J. Nat. Prod. 69, 1572–1576. 10.1021/np0601861 17125223

[B89] Namkoong S. Kim T. J. Jang I. S. Kang K. W. Oh W. K. Park J. (2011). Alpinumisoflavone induces apoptosis and suppresses extracellular signal-regulated kinases/mitogen activated protein kinase and Nuclear Factor-κB pathways in lung tumor cells. Biol. Pharm. Bull. 34, 203–208. 10.1248/bpb.34.203 21415528

[B90] Namouchi A. Cimino M. Favre-Rochex S. Charles P. Gicquel B. (2017). Phenotypic and genomic comparison of *Mycobacterium aurum* and surrogate model species to *Mycobacterium tuberculosis*: implications for drug discovery. BMC Genomics 18, 530. 10.1186/s12864-017-3924-y 28705154 PMC5508667

[B91] Nana F. Sandjo L. S. Keumedjio F. Ambassa P. Malik R. Kuete V. (2012). Ceramides and cytotoxic constituents from *Ficus glumosa* Del. (Moraceae). J. Braz. Chem. Soc. 23, 482–487. 10.1590/S0103-50532012000300015

[B92] Naoi M. Maruyama W. Shamoto-Nagai M. (2018). Type A and B monoamine oxidases distinctly modulate signal transduction pathway and gene expression to regulate brain function and survival of neurons. J. Neural Transm. 125, 1635–1650. 10.1007/s00702-017-1832-6 29279995

[B93] Ncube N. S. Afolayan A. J. Okoh A. I. (2008). Assessment techniques of antimicrobial properties of natural compounds of plant origin: current methods and future trends. Afr. J. Biotechnol. 7, 1797–1806. 10.5897/AJB07.613

[B94] Ndemangou B. Tedjon Sielinou V. Vardamides J. C. Shaiq Ali M. Lateef M. Iqbal L. (2013). Urease inhibitory isoflavonoids from different parts of *Calopogonium mucunoides* (Fabaceae). J. Enzym. Inhib. Med. Chem. 28 (6), 1156–1161. 10.3109/14756366.2012.719025 23057815

[B95] Nkengfack A. E. Azebaze A. G. B. Waffo A. K. Fomum Z. T. Meyer M. Van Heerden F. R. (2001). Cytotoxic isoflavones from *Erythrina indica*. Phytochemistry 58, 1113–1120. 10.1016/S0031-9422(01)00368-5 11730876

[B96] Nwodo J. N. Ibezim A. Simoben C. V. Ntie-Kang F. (2016). Exploring cancer therapeutics with natural products from African medicinal plants, Part II: alkaloids, terpenoids and flavonoids. Anti-Cancer Agent. Med. Chem. 16, 108–127. 10.2174/1871520615666150520143827 25991425

[B97] Nyandoro S. S. Munissi J. J. E. Kombo M. Mgina C. A. Pan F. Gruhonjic A. (2017). Flavonoids from *Erythrina schliebenii*. J. Nat. Prod. 80, 377–383. 10.1021/acs.jnatprod.6b00839 28112509

[B98] Oakley R. H. Cidlowski J. A. (2013). The biology of the glucocorticoid receptor: new signaling mechanisms in health and disease. J. Allergy Clin. Immun. 132, 1033–1044. 10.1016/j.jaci.2013.09.007 24084075 PMC4084612

[B99] Ocloo A. Kingsford-Adaboh R. Murray A. J. (2017). Inhibition of mitochondrial respiratory chain activity by O, O-dimethyl- and 4-O-methyl-Alpinumisoflavones. J. Appl. Pharm. Sci. 7, 95–100.

[B100] Oh W. K. Lee C. H. Seo J. H. Chung M. Y. Cui L. Fomum Z. T. (2009). Diacylglycerol acyltransferase-inhibitory compounds from *Erythrina senegalensis*. Arch. Pharm. Res. 32, 43–47. 10.1007/s12272-009-1116-2 19183875

[B101] Oh W. K. Kim B. Y. Oh H. Kim B. S. Ahn J. S. (2005). Phospholipase Cγ1 inhibitory activities of prenylated flavonoids isolated from *Erythrina senegalensis*. Planta Med. 71, 780–782. 10.1055/s-2005-864183 16142647

[B102] Okamoto Y. Suzuki A. Ueda K. Ito C. Itoigawa M. Furukawa H. (2006). Anti-estrogenic activity of prenylated isoflavones from *Millettia pachycarpa*: implications for pharmacophores and unique mechanisms. J. Health Sci. 52 (2), 186–191. 10.1248/jhs.52.186

[B103] Patil V. M. Masand N. (2019). Anticancer potential of flavonoids: chemistry, biological activities, and future perspectives. Stud. Nat. Prod. Chem. 59, 401–430. 10.1016/B978-0-444-64179-3.00012-8

[B104] Perese D. A. Ulman J. Viola J. Ewing S. E. E. Bankiewicz K. S. (1989). A 6-Hydroxydopamine-induced selective parkinsonian rat model. Brain Res. 494, 285–293. 10.1016/0006-8993(89)90597-0 2528389

[B105] Power O. Jakeman P. FitzGerald R. J. (2013). Antioxidative peptides: enzymatic production, *in vitro* and *in vivo* antioxidant activity and potential applications of milk-derived antioxidative peptides. Amino Acids 44, 797–820. 10.1007/s00726-012-1393-9 22968663

[B106] Prior R. L. Wu X. Schaich K. (2005). Standardized methods for the determination of antioxidant capacity and phenolics in foods and dietary supplements. J. Agric. Food Chem. 53, 4290–4302. 10.1021/jf0502698 15884874

[B107] Rahman M. Z. Rahman M. S. Kaisar A. Hossain A. Rashid M. A. (2010). Bioactive isoflavones from *Erythrina variegata* L. Turk. J. Pharm. Sci. 7, 21–28.

[B108] Rani K. (2017). Role of antioxidants in prevention of diseases. J. Appl. Biotechnol. Bioeng. 4 (1), 00091. 10.15406/jabb.2017.04.00091

[B109] Reynaud J. Guilet D. Terreux R. Lussignol M. Walchshofer N. (2005). Isoflavonoids in non-leguminous families: an update. Nat. Prod. Rep. 22, 504–515. 10.1039/b416248j 16047048

[B110] Riaz N. Akram Naveed M. Saleem M. Jabeen B. Ashraf M. Ejaz S. A. (2012). Cholinesterase inhibitory constituents from *Ficus bengalensis*. J. Asian Nat. Prod. Res. 14, 1149–1155. 10.1080/10286020.2012.733702 23106601

[B111] Ríos J. L. Recio M. C. (2005). Medicinal plants and antimicrobial activity. J. Ethnopharmacol. 100, 80–84. 10.1016/j.jep.2005.04.025 15964727

[B112] Rohwer N. Cramer T. (2011). Hypoxia-mediated drug resistance: novel insights on the functional interaction of HIFs and cell death pathways. Drug Resist. Updat. 14, 191–201. 10.1016/j.drup.2011.03.001 21466972

[B113] San-Martín A. Donoso V. Leiva S. Bacho M. Núñez S. Gutierrez M. (2015). Molecular docking studies of the antitumoral activity and characterization of new chalcone. Curr. Top. Med. Chem. 15, 1743–1749. 10.2174/1568026615666150427125033 25915607

[B114] Sathishkumar R. Tharani R. (2017). *In silico* determination of efficiency of plant secondary metabolites to eradicate Trachoma-A blinding keratoconjuctivitis disease. J. Appl. Pharm. Sci. 7, 116–121.

[B115] Schito L. Semenza G. L. (2016). Hypoxia-inducible factors: master regulators of cancer progression. Trends Cancer 2, 758–770. 10.1016/j.trecan.2016.10.016 28741521

[B116] Schober A. (2004). Classic toxin-induced animal models of Parkinson’s disease:6-OHDA and MPTP. Cell Tissue Res. 318, 215–224. 10.1007/s00441-004-0938-y 15503155

[B117] Sharma O. P. Bhat T. K. (2009). DPPH antioxidant assay revisited. Food Chem. 113, 1202–1205. 10.1016/j.foodchem.2008.08.008

[B118] Shen B. (2015). A new golden age of natural products drug discovery. Cell 163, 1297–1300. 10.1016/j.cell.2015.11.031 26638061 PMC5070666

[B119] Sherif S. H. Vidavalur S. Muralidhar P. Murthy Y. L. N. (2015). Synthesis and antioxidant activities of naturally occurring alpinum isoflavone, 4′-O-methylalpinum isoflavone and their synthetic analogues. Der Pharma Chemica 7 (5), 116–123.

[B120] Shi L. (2010). Separation, purification and structure characterization of a polysaccharide from root of *Cudrania tricuspidata*. Asian J. Exp. Biol. Sci. 1, 311–314.

[B121] Shin G. R. Lee S. Lee S. Do S. G. Shin E. Lee C. H. (2015). Maturity stage-specific metabolite profiling of *Cudrania tricuspidata* and its correlation with antioxidant activity. Ind. Crop. Prod. 70, 322–331. 10.1016/j.indcrop.2015.01.048

[B122] Su Z. Q. Mo Z. Z. Liao J. B. Feng X. X. Liang Y. Z. Zhang X. (2014). Usnic acid protects LPS-induced acute lung injury in mice through attenuating inflammatory responses and oxidative stress. Int. Immunopharmacol. 22, 371–378. 10.1016/j.intimp.2014.06.043 25068825

[B123] Sudanich S. Tiyaworanant S. Yenjai C. (2017). Cytotoxicity of flavonoids and isoflavonoids from *Crotalaria bracteata*. Nat. Prod. Res. 31, 2641–2646. 10.1080/14786419.2017.1289207 28278675

[B124] Tan J. B. L. Lim Y. Y. (2015). Critical analysis of current methods for assessing the *in vitro* antioxidant and antibacterial activity of plant extracts. Food Chem. 172, 814–822. 10.1016/j.foodchem.2014.09.141 25442625

[B125] Tanaka Y. (2014). Glucocorticoid and bone. Pathogenesis of glucocorticoid-induced osteoporosis. Clin. Calcium 24, 1289–1294. 25177000

[B126] Tjahjandarie T. S. Tanjung M. (2015a). Phenolic compounds from the stem bark of *Erythrina orientalis* and their cytotoxic and antioxidant activities. Der Pharma Chemica 7 (1), 206–211.

[B127] Tjahjandarie T. S. Tanjung M. (2015b). Antiplasmodial isoprenylated flavonoids from the stem bark of *Erythrina ovalifolia* Roxb. Der Pharmacia Lettre 7 (2), 35–39.

[B128] Tong J. Rathitharan G. Meyer J. H. Furukawa Y. Ang L. C. Boileau I. (2017). Brain monoamine oxidase B and A in human parkinsonian dopamine deficiency disorders. Brain 140, 2460–2474. 10.1093/brain/awx172 29050386 PMC6669411

[B129] Trinh B. T. D. Jäger A. K. Staerk D. (2017). High-resolution inhibition profiling combined with HPLC-HRMS-SPE-NMR for identification of PTP1B inhibitors from Vietnamese Plants. Molecules 22, 1228. 10.3390/molecules22071228 PMC6152321 28726759

[B130] Uddin M. M. N. Emran T. B. Mahib M. M. R. Dash R. (2014). Molecular docking and analgesic studies of *Erythrina variegata*‘s derived phytochemicals with COX enzymes. Bioinformation 10 (10), 630–636. 10.6026/97320630010630 25489172 PMC4248345

[B131] van de Laar F. A. Lucassen P. L. Akkermans R. P. de Lisdonk E. H. Rutten G. E. Van Weel C. (2005). Glucosidase inhibitors for patients with type 2 diabetes. Diabetes Care 28, 166–175. 10.2337/diacare.28.7.1841 15616251

[B132] van Tonder A. Joubert A. M. Cromarty A. D. (2015). Limitations of the 3-(4,5-dimethylthiazol-2-yl)-2,5-diphenyl-2H-tetrazolium bromide (MTT) assay when compared to three commonly used cell enumeration assays. BMC Research Notes 8, 47. 10.1186/s13104-015-1000-8 25884200 PMC4349615

[B133] Venturelli S. Burkard M. Biendl M. Lauer U. M. Frank J. Busch C. (2016). Prenylated chalcones and flavonoids for the prevention and treatment of cancer. Nutrition 32, 1171–1178. 10.1016/j.nut.2016.03.020 27238957

[B134] Waffo A. K. Azebaze G. A. Nkengfack A. E. Fomum Z. T. Meyer M. Bodo B. (2000). Indicanines B and C, two isoflavonoid derivatives from the root bark of *Erythrina indica*. Phytochemistry 53, 981–985. 10.1016/S0031-9422(99)00615-9 10820816

[B135] Walle T. Ta N. Kawamori T. Wen X. Tsuji P. A. Walle U. K. (2007). Cancer chemopreventive properties of orally bioavailable flavonoids-methylated versus unmethylated flavones. Biochem. Pharmacol. 73, 1288–1296. 10.1016/j.bcp.2006.12.028 17250812 PMC1868573

[B136] Wang L. J. Jiang B. Wu N. Wang S. Y. Shi D. Y. (2015). Natural and semisynthetic protein tyrosine phosphatase 1B (PTP1B) inhibitors as anti-diabetic agents. RSC Adv. 5, 48822–48834. 10.1039/C5RA01754H

[B137] Wang S. Y. Sun Z. L. Liu T. Gibbons S. Zhang W. J. Qing M. (2014). Flavonoids from *Sophora moorcroftiana* and their synergistic antibacterial effects on MRSA. Phytother. Res. 28, 1071–1076. 10.1002/ptr.5098 24338874

[B138] Wang T. Jiang Y. Chu L. Wu T. You J. (2017a). Alpinumisoflavone suppresses tumour growth and metastasis of clear-cell renal cell carcinoma. Am. J. Cancer Res. 7, 999–1015. 28469971 PMC5411806

[B139] Wang Y. Liu J. Pang Q. Tao D. (2017b). Alpinumisoflavone protects against glucocorticoid-induced osteoporosis through suppressing the apoptosis of osteoblastic and osteocytic cells. Biomed. Pharmacother. 96, 993–999. 10.1016/j.biopha.2017.11.136 29203387

[B140] Wang T. Liu Y. Li X. Xu Q. Feng Y. Yang S. (2018). Isoflavones from green vegetable soya beans and their antimicrobial and antioxidant activities. J. Sci. Food Agric. 98, 2043–2047. 10.1002/jsfa.8663 28885710

[B141] Wang P. Henning S. M. Heber D. (2010). Limitations of MTT and MTS-based assays for measurements of antiproliferative activity of green tea polyphenols. PLoS One 5 (4), e10202. 10.1371/journal.pone.0010202 20419137 PMC2855713

[B142] WHO (2017). Global Tuberculosis Report 2017. Geneva: World Health Organization.

[B143] Xin L. T. Yue S. J. Fan Y. C. Wu J. S. Yan D. Guan H. S. (2017). *Cudrania tricuspidata*: an updated review on ethnomedicine, phytochemistry and pharmacology. RSC Adv. 7, 31807–31832. 10.1039/C7RA04322H

[B144] Xiong W. Y. Wang J. Z. Shi T. D., (1993). Woody medicine plants of China. Shanghai: Shanghai Science and Education Press, Shanghai, 85–88.

[B145] Yang X. Jiang Y. Yang J. He J. Sun J. Chen F. (2015). Prenylated flavonoids, promising nutraceuticals with impressive biological activities. Trends Food Sci. Tech. 44, 93–104. 10.1016/j.tifs.2015.03.007

[B146] Yeh C. H. Yang J. J. Yang M. L. Li Y. C. Kuan Y. H. (2014). Rutin decreases lipopolysaccharide-induced acute lung injury *via* inhibition of oxidative stress and the MAPK-NF-k B pathway. Free Radical Biol. Med. 69, 249–257. 10.1016/j.freeradbiomed.2014.01.028 24486341

[B147] Yin J. Han L. Cong W. (2018). Alpinumisoflavone rescues glucocorticoid-induced apoptosis of osteocytes *via* suppressing Nox2-dependent ROS generation. Pharmacol. Rep. 70, 270–276. 10.1016/j.pharep.2017.11.001 29477034

[B148] Zhang B. Fan X. Wang Z. Zhu W. Li J. (2017). Alpinumisoflavone radiosensitizes esophageal squamous cell carcinoma through inducing apoptosis and cell cycle arrest. Biomed. Pharmacother. 95, 199–206. 10.1016/j.biopha.2017.08.048 28843908

[B149] Zhang M. Qiu Q. Li Z. Sachdeva M. Min H. Cardona D. M. (2015). HIF-1α regulates the response of primary sarcomas to radiation therapy through a cell autonomous mechanism. Radiat. Res. 183, 594–609. 10.1667/RR14016.1 25973951 PMC4800000

[B150] Zheng Z. P. Tan H. Y. Chen J. Wang M. (2013). Characterization of tyrosinase inhibitors in the twigs of *Cudrania tricuspidata* and their structure–activity relationship study. Fitoterapia 84, 242–247. 10.1016/j.fitote.2012.12.006 23262271

